# Heart rate variability in different sleep stages is associated with metabolic function and glycemic control in type 2 diabetes mellitus

**DOI:** 10.3389/fphys.2023.1157270

**Published:** 2023-04-14

**Authors:** Wenquan Cheng, Hongsen Chen, Leirong Tian, Zhimin Ma, Xingran Cui

**Affiliations:** ^1^ State Key Laboratory of Bioelectronics, School of Biological Science and Medical Engineering, Southeast University, Nanjing, China; ^2^ Endocrinology Department, Suzhou Science and Technology Town Hospital, Suzhou, China; ^3^ Institute of Medical Devices (Suzhou), Southeast University, Suzhou, China

**Keywords:** T2DM, heart rate variability (HRV), sleep, autonomic nervous system (ANS), metabolic function, nonlinear analysis, glycemic control

## Abstract

**Introduction:** Autonomic nervous system (ANS) plays an important role in the exchange of metabolic information between organs and regulation on peripheral metabolism with obvious circadian rhythm in a healthy state. Sleep, a vital brain phenomenon, significantly affects both ANS and metabolic function.

**Objectives:** This study investigated the relationships among sleep, ANS and metabolic function in type 2 diabetes mellitus (T2DM), to support the evaluation of ANS function through heart rate variability (HRV) metrics, and the determination of the correlated underlying autonomic pathways, and help optimize the early prevention, post-diagnosis and management of T2DM and its complications.

**Materials and methods:** A total of 64 volunteered inpatients with T2DM took part in this study. 24-h electrocardiogram (ECG), clinical indicators of metabolic function, sleep quality and sleep staging results of T2DM patients were monitored.

**Results:** The associations between sleep quality, 24-h/awake/sleep/sleep staging HRV and clinical indicators of metabolic function were analyzed. Significant correlations were found between sleep quality and metabolic function (|r| = 0.386 ± 0.062, *p* < 0.05); HRV derived ANS function showed strengthened correlations with metabolic function during sleep period (|r| = 0.474 ± 0.100, *p* < 0.05); HRV metrics during sleep stages coupled more tightly with clinical indicators of metabolic function [in unstable sleep: |r| = 0.453 ± 0.095, *p* < 0.05; in stable sleep: |r| = 0.463 ± 0.100, *p* < 0.05; in rapid eye movement (REM) sleep: |r| = 0.453 ± 0.082, *p* < 0.05], and showed significant associations with glycemic control in non-linear analysis [fasting blood glucose within 24 h of admission (admission FBG), |r| = 0.420 ± 0.064, *p* < 0.05; glycated hemoglobin (HbA1c), |r| = 0.417 ± 0.016, *p* < 0.05].

**Conclusions:** HRV metrics during sleep period play more distinct role than during awake period in investigating ANS dysfunction and metabolism in T2DM patients, and sleep rhythm based HRV analysis should perform better in ANS and metabolic function assessment, especially for glycemic control in non-linear analysis among T2DM patients.

## 1 Introduction

Type 2 diabetes mellitus (T2DM) is a chronic hyperglycemia that causes physiological dysfunction and failure of various organs. Autonomic neuropathy is one of the most common complications of diabetes mellitus, which seriously affects the patients’ life quality and brings about significant morbidity and mortality (Maser et al., 2003; Vinik et al., 2003; Ziegler et al., 2008). The dysfunction of autonomic nervous system (ANS) usually manifests first in vagal nerve (the longest parasympathetic nerve in the body, responsible for nearly three-quarters of parasympathetic activity), and damage to vagal nerve leads to resting tachycardia and an overall decrease in parasympathetic tone (Balcıoğlu and Müderrisoğlu, 2015).

Dysfunction of ANS, caused by T2DM, happens in any part of ANS from early stages of diabetes, which damages the cardiovascular, gastrointestinal, genitourinary and neurovascular systems (Pfeifer et al., 1982; Singh et al., 2000; Goit et al., 2012). The function of healthy ANS has obvious circadian rhythm (Cui et al., 2020), and the dysfunction of ANS can result in the loss of circadian rhythm to varying degrees (Liu et al., 2020). Consequently, diabetes is often followed by sleep disorders (Barone and Menna-Barreto, 2011). Meanwhile, sleep disorders accelerate the development of T2DM by worsening the metabolic control, which forms into a vicious spiral (Barone and Menna-Barreto, 2011). Researchers have found that poor sleep quality (Martyn-Nemeth et al., 2018), insufficient or excessive sleep duration, changes of sleep structure (Koren et al., 2011), decreasing sleep efficiency (Hur et al., 2020), increasing OSA severity (Kent et al., 2014), etc. are associated with poor diabetic control. Also, sleep disorders are strongly related to ANS function of T2DM patients (Jordan et al., 2014). The activation of the sympathetic nervous system (SNS) and the unbalance of ANS in patients with T2DM could be mediated through sleep impairments, including nocturnal breathing disturbances (Punjabi and Polotsky, 2005; López-Cano et al., 2019) and sleep curtailment (Leproult et al., 1997; Spiegel et al., 1999).

Heart rate variability (HRV) is considered to be an effective measure of heart-brain interaction and tension of ANS (Montano et al., 2009), and is widely used as a standard method for assessing autonomic function. Studies have found that T2DM patients’ HRV differs significantly from normal people in time-domain, frequency-domain and non-linear analysis (Barone and Menna-Barreto, 2011; Faust et al., 2012; Martyn-Nemeth et al., 2018; Liu et al., 2020). Therefore, HRV analysis is considered an effective, non-invasive auxiliary detection method for T2DM patients’ autonomic dysfunction. Cardiologists tend to analyze 24-h HRV, whereas internists tend to detect whether HRV is abnormal in different physiological states, known as Ewing test (Gerritsen et al., 2001).

Despite the full utilization in clinical fields, HRV analysis in current researches mainly focused on linear analysis of 24-h or 5–15 min electrocardiogram (ECG) collected in different physiological states, which fails to effectively extract the information in long-term signals and neglects the space-time complexity and fractal properties of heart rate time series. Meanwhile, it was a general problem that HRV metrics were accepted as measures of autonomic function without examination of the underlying physiological patterns (Stein and Pu, 2012) and autonomic pathways. Hence, controversial research results were presented. For example, researchers have found that low-frequency component of HRV was not predominantly influenced by SNS, but vagal nerve system (Reyes del Paso et al., 2013), and reflected multiple sources of variability (Martelli et al., 2014), which also challenged the usefulness of the ratio of low-frequency component to high-frequency component (LF/HF); higher HRV were related to disturbed sleep, such as in sleep disorders as negative feature (Stein and Pu, 2012); imparity of HRV metrics reflecting parasympathetic nervous system (PNS), such as high-frequency power (HF) and root mean square of successive of RR interval differences (RMSSD), occurred during different sleep stages (Glos et al., 2014; Zhang et al., 2020).

Therefore, based on the complex associations among sleep, ANS and T2DM, we monitored 24-h ECG, clinical metabolic indicators, sleep quality as well as sleep staging results of T2DM patients. Our objective was to help optimize the early prevention, post-diagnosis and management of T2DM and its complications. Our hypothesis included: 1) HRV derived ANS function is more associated with T2DM patients’ metabolic function during sleep than during awake or the whole 24-h; 2) ANS function alters during sleep cycles, leading to changes on its interaction with metabolic system, thus we analyzed interaction between HRV derived ANS function with metabolic function in each sleep stage; finally, 3) non-linear HRV analysis during sleep cycles may discover more features of ANS function than conventionally used linear HRV analysis, leading to more extensive association with metabolic function and glycemic control.

## 2 Materials and methods

### 2.1 Patients

The study was conducted form July 2019 to January 2021. A total of 64 volunteered inpatients with T2DM from Suzhou Science & Technology Town Hospital took part in this study. Exclusion criteria were: type 1 diabetes mellitus, history of stroke, subacute myocardial infarction, kidney or liver transplant, other systemic disorders, current recreational drug or alcohol abuse and morbid obesity (body mass index >40). Because of the small number of female inpatients in the study (only 4), and considering that women in the same age group may be affected metabolically by perimenopausal syndrome, and decreasing the bias brought by sex (Nunan et al., 2010), data from only 60 male inpatients were included in the follow-up studies. This study was approved by the Institutional Review Board of Suzhou Science & Technology Town Hospital (No. IRB2019045). Prior to the study, all subjects were informed of the experimental protocol and precautions, and signed the written informed consent. Demographic characteristics of the subjects are demonstrated in Table 1. The Strengthening the Reporting of Observational Studies in Epidemiology (STROBE) guidelines for cross-sectional studies were followed for study reporting (Cuschieri, 2019).

**TABLE 1:** Characteristics of the study cohort. Data are presented as mean ± standard deviation (SD) if the variable is normally distributed, otherwise presented as median (p25, p75).

Parameters (Unit)	Data	Sample size
*Demographics*		
Age (years)	50 ± 16	60
Weight (kg)	77.5 ± 13.9	60
Height (cm)	172 ± 6	60
*Clinical Indicators of Metabolic Function*		
Admission FBG (mmol/L)	9.1 ± 3.1	52
Discharge FBG (mmol/L)	6.5 (5.2, 6.8)	37
HbA1c (%)	8.2 ± 1.7	40
SBP (mmHg)	136 ± 14	58
DBP (mmHg)	85 ± 11	58
WBC (×10^9^/L)	5.5 (5.0, 6.4)	56
N% (%)	62.8 ± 10.9	56
Hb (g/L)	146 ± 17	56
PLT (×10^9^/L)	203 ± 56	56
CRP (mg/L)	3 (1, 4)	51
ALT (U/L)	22 (11, 45)	56
AST (U/L)	19 (13, 23)	55
AST/ALT	0.867 (0.613, 1.188)	55
GGT (U/L)	25 (14, 49)	56
BUN (mmol/L)	5.6 ± 1.7	50
UA (μmol/L)	340 ± 102	51
TG (mmol/L)	1.14 (0.86, 1.64)	52
HDL-C (mmol/L)	1.02 ± 0.24	51
LDL-C (mmol/L)	2.50 ± 0.77	51
UMA (mg)	15.90 (6.05, 22.10)	49
UCr (g)	10.38 ± 5.90	47
UACR (mg/g)	1.097 (0.751, 1.853)	47
*Diabetic Complications (n/total, n%)*		
Diabetic nephropathy	6/58, 10.34%	
Diabetic retinopathy and cataract	16/58, 27.59%	
Diabetic peripheral neuropathy	4/58, 6.90%	
Coronary artery disease and cardiac insufficiency	3/58, 5.17%	
Lower extremity atherosclerosis or stenosis	4/58, 6.90%	
Carotid plaque	10/58, 17.24%	
Any of above complications	29/58, 50.00%	

Admission FBG, fasting blood glucose within 24 h of admission; Discharge FBG, fasting blood glucose within 24 h before discharge; HbA1c, glycated hemoglobin; SBP, systolic blood pressure; DBP, diastolic blood pressure; WBC, white blood cell; N%, percent of the number of neutrophils in the number of white blood cells; Hb, hemoglobin; PLT, platelet; CRP, c-reactive protein; ALT, alanine aminotransferase; AST, aspartate aminotransferase; AST/ALT, ratio of AST to ALT; GGT, gamma glutamyltransferase; BUN, blood urea nitrogen; UA, uric acid; TG, triglycerides; HDL-C, high-density lipoprotein-cholesterol; LDL-C, low-density lipoprotein-cholesterol; UMA, urine micro-albumin; UCr, urine creatinine; UACR, urine albumin-creatinine ratio.

### 2.2 Clinical indicators of metabolic function

During hospitalization, subjects underwent clinical examinations to obtain their metabolic function and assess their health status. Upon admission, a fasting blood draw and urinary sample were obtained the next morning for routine glucose, lipid and renal panels. Subjects were also involved in investigation for diabetic complications. Clinical indicators of metabolic function analyzed in this study and diabetic complications statistics are demonstrated in Table 1.

Subsequently, clinical indicator outlier rejection was performed where box plot was utilized. The data were rearranged from largest to smallest, with the difference between the upper quartile U and the lower quartile L defined as IQR. The upper bound was set to U+1.5*IQR and the lower bound was set to L-1.5*IQR, and data exceeding the upper and lower bounds can be considered as outliers. Nevertheless, some data that did not appear to be gross errors, i.e., that were outside the range of normal values but might appear in patients who are in dysfunction states, were retained with the aim of reserving more information underneath the pathological data.

An HbA1c level greater than 9.0% indicates poor control of diabetes, which corresponds to a greater risk of complications and the need for more stringent treatment strategies (Inzucchi et al., 2015; Mosenzon et al., 2016; Ye et al., 2016; Garber et al., 2020). Subjects could be assigned to two groups according to the HbA1c level: one group had poor control of diabetes (HbA1c ≥ 9.0%) and the other did not (HbA1c < 9.0%).

### 2.3 ECG data recording

ECG recordings were collected by an FDA (U.S. Food and Drug Administration) approved ambulatory electrocardiogram monitor (DynaDx Corporation, Mountainview, CA, United States) with a computer-based data-acquisition system. The ECG recording equipment was a single-lead Holter device that can record ECG for over 24 h. Sampling frequency of ECG monitoring was set to 250 Hz. All subjects were monitored in hospital for 24-h, starting at 10 p.m. on the second day of hospitalization. The 24-h ECG data was analyzed for HRV, which will be reported elsewhere. All ECG recordings were carefully checked with noise level, artifacts, and ectopic beats. Seventeen ECG recordings were discarded due to low data quality, too short recording time, or the fact that the inpatients had atrial fibrillation or server arrhythmia.

### 2.4 ECG data preprocessing

The band-pass Butterworth filter and zero-phase shift filter with a cut-off frequency of 5 Hz–35 Hz were first used to eliminate the baseline drift, power frequency interference, myoelectric interference, motion artifacts and equipment noise.

At present, plenty of R-peak detection methods have been proposed on ECG signal analysis. However, when applying these detectors on ECG signals collected in long-term recordings especially *via* wearable single-lead ECG devices, the R-peak detection accuracies were usually unsatisfying. To guarantee the accuracy of R-peak detection, a method for extracting high-quality RR intervals proposed in our previous study (Cui et al., 2020) was applied, which combing five commonly used R-peak detection methods including “Pan-Tompkins” (Pan and Tompkins, 1985; Hamilton and Tompkins, 1986), “jqrs” (Johnson et al., 2015), “mteo” (Solnik et al., 2008), “nqrs” (Li and Yang, 2013), and “sixth power” (Dohare et al., 2014). Outlier rejection for RR interval time series was performed, and box plot was utilized to find and reject outliers.

### 2.5 HRV analysis and metrics

In this study, we evaluated HRV based on the widely-used linear and non-linear HRV metrics (Task Force of the European Society of Cardiology the North American Society of Pacing Electrophysiology, 1996; Sassi et al., 2015).

#### 2.5.1 Linear HRV analysis

Linear HRV metrics include time-domain metrics and frequency-domain metrics. Time-domain metrics quantify the amount of variability of the time period between successive heartbeats (RR intervals), and include HR-mean (mean of heart rate), SDNN (standard deviation of RR intervals), RMSSD (root mean square of successive RR intervals differences), SDSD (standard deviation of successive RR interval differences), SDANN (the standard deviation of the average value every 5 min in the RR interval) and pNN50 (the percentage of successive RR intervals that differ by more than 50 ms). Frequency-domain metrics estimate the distribution of absolute or relative power at different frequency bands. Generally, the whole frequency band (whose absolute power is noted as TP) is divided into three frequency bands: high-frequency band (0.15–0.40 Hz, whose absolute power is noted as HF), low-frequency band (0.04–0.15 Hz, whose absolute power is noted as LF) and very-low-frequency band (0.00–0.04 Hz, whose absolute power is noted as VLF). Other frequency-domain metrics include LFP (percentage of LF in TP), HFP (percentage of HF in TP) and LF/HF (ratio of LF-to-HF power). The widely used HRV analysis model for assessing ANS homeostasis includes three core statements (Pagani et al., 1986; Malliani et al., 1991; Montano et al., 2009): 1) the power of the high-frequency component reflects cardiac parasympathetic activity; 2) the power of low-frequency component reflects cardiac sympathetic activity; and 3) the LF-HF-ratio reflects sympathetic parasympathetic balance.

#### 2.5.2 Non-linear HRV analysis

Non-linear analysis utilized in this study includes approximate entropy (ApEn) (Pincus, 1991), sample entropy (SampEn) (Chen et al., 2009), fuzzy entropy (FuzzyEn) (Richman and Moorman, 2000; Azami and Escudero, 2016), detrended fluctuation analysis (DFA, alpha1 and alpha2) (Peng et al., 1994), multi-scale entropy (MSE) (Costa et al., 2002) and multi-scale fuzzy entropy (MFE). Calculation steps are introduced in the supplementary material.

ApEn is a rather conventional measure to quantify irregularity and complexity and reflects the probability of new subsequences, and the more complex time series corresponds to larger ApEn. SampEn is a modification of ApEn, and is more accurate than ApEn in the case of less data. FuzzyEn is improved on the basis of SampEn, which is the entropy of a fuzzy set that contains vagueness and ambiguity uncertainties. When calculating ApEn, the embedding dimension m was set to 2, and the similar tolerance r was set to 0.25 times the SD of the sequence in order to find significant difference more clearly; in SampEn and FuzzyEn, r was set to 0.15 times the SD of the sequence.

MSE analyzes the complexity of time series from multiple time scales. The 25 coarse-grained time series are contracted from the original RR time series, in which we obtained 28 indices, including the SampEn value at each coarse-graining scale and 3 complexity metrics, MSEsum5, MSEsum10 and MSEsum20 (defined as the area of the MSE curve at scales 1–5, 1–10 and 1–20 respectively). The MSE curve usually rises rapidly at lower scales, peaks at scale 5, and then declines slowly, reaching a plateau after scale 20, therefore calculating the area of the MSE curve at scales 1–5 and 1–20 can portray the complexity characteristics of this curve at high and low resolutions. Similarly, MFE were calculated based on FuzzyEn.

DFA is a non-linear fractal analysis tool to discover potential self-similarity in the series and quantify the fractal scale of the time series. The α1 and α2 portray the short- and long-range correlation respectively.

### 2.6 Sleep quality assessment

Subjective sleep quality was assessed by Pittsburgh Sleep Quality Index (PSQI) and a brief sleep log was used to record sleep duration for the studied night. PSQI includes multiple sleep-related variables over the preceding month, using Likert and open-ended response formats (Spira et al., 2011). The PSQI yields seven component scores: subjective sleep quality, sleep latency, sleep duration, habitual sleep efficiency, sleep disturbances, sleep medication, and daytime dysfunction. Component scores range from 0 to 3 and are summed to obtain a global score, which ranges from 0 to 21. Higher scores suggest greater sleep disturbance (Buysse et al., 1989). During 24-h ECG monitoring, 53 subjects filled in PSQI questionnaire.

Objective sleep quality and sleep staging were assessed by ECG-based cardiopulmonary coupling (CPC) analysis (Thomas et al., 2005), which has been cited in more than 260 publications (Yeh et al., 2008; Schramm et al., 2009; Thomas et al., 2010), and was introduced in the famous international sleep monograph “Principle and Practice of Medicine” (the sixth edition). The ECG recordings during sleep at night were extracted for sleep analysis in this study. CPC analysis is based on mathematical analysis of the coupling between HRV and the respiratory modulation of QRS waveform on a beat-to-beat basis. Major physiological sleep states derived from CPC analysis include stable sleep (indicated by high-frequency coupling, or HFC), unstable sleep (indicated by low frequency coupling, or LFC), and rapid eye movement (REM) or wakeful states (indicated by very low frequency coupling, or VLFC) (Thomas et al., 2005). In this study, 38 CPC sleep reports were available. Sleep quality metrics analyzed in this study are demonstrated in Table 2.

**TABLE 2:** Sleep quality metrics analyzed in this study. Data are presented as mean ± SD if the variable is normally distributed, otherwise presented as median (p25, p75).

Metrics (Unit)	Data	Sample size
PSQI	6 ± 3	53
AHI (event per hour)	21.2 (12.5, 38.8)	38
TST (hour)	7.8 ± 1.5	38
UST (hour)	3.8 ± 1.6	38
SST (hour)	2.5 ± 1.2	38
RST (hour)	1.6 ± 0.7	38
ALUS (min)	8.4 (6.4, 12.8)	38
ALSS (min)	11.2 ± 4.2	38
ALRS (min)	4.7 ± 1.1	38
USP (%)	47.53 ± 17.45	38
SSP (%)	32.22 ± 15.38	38
RSP (%)	20.45 ± 7.81	38

PSQI, Pittsburgh sleep quality index; AHI, apnea-hypopnea index; TST, total sleep time; UST, unstable sleep time; SST, stable sleep time; RST, rapid eye movement sleep time; ALUS, average length of unstable sleep segments; ALSS, average length of stable sleep segments; ALRS, average length of rapid eye movement sleep segments; USP, percentage of unstable sleep time in total sleep time; SSP, percentage of stable sleep time in total sleep time; RSP, percentage of rapid eye movement sleep time in total sleep time.

### 2.7 Statistical analysis

The data were analyzed by SPSS version 27.0 for Windows. The statistical analysis included significance analysis and correlation analysis. The normality of distribution of continuous variables was firstly tested by the Shaprio-Wilk test (significance level *α* = 0.05). For two normally distributed continuous variables, the Pearson correlation analysis was utilized to estimate the level of correlation, otherwise the Spearman analysis. Partial correlation analysis was also involved in the assessment of correlations between variables. For normally distributed variables, group differences were compared by independent sample *t*-test (2 groups) and one-way ANOVA (3 or more groups), otherwise Mann-Whitney U test (2 groups) or Kruskal-Wallis test (3 or more groups) were used. A value of *p* < 0.05 was considered significant. Missing data was completely at random, and pairwise deletion was conducted during the statistical analyses.

There are 25 scales and 3 complexity metrics for MSE and MFE respectively, and in order to demonstrate their overall correlations with clinical indicators in a straightforward way, we define the correlations between MSE and clinical indicators as mean ± SD of the correlations between SampEn at specific scales and clinical indicators. The coefficients of the same significant level (*p* < 0.05 or *p* < 0.01) between SampEn at certain scale and clinical indicators were selected to calculate the mean and SD. Similarly, the correlations between MFE and clinical indicators can be calculated. As for complexity metrics, the correlations between MSE complexity and clinical indicators are defined as mean ± SD of the correlations between specific complexity metrics (MSEsum5, MSEsum10 and MSEsum20) of the same significant level and clinical indicators, and similarly the correlations between MFE complexity and clinical indicators could be calculated.

## 3 Results

### 3.1 Correlations between sleep quality metrics and clinical indicators

Table 3 demonstrates the correlation coefficients between sleep quality metrics and clinical indicators. PSQI correlated rather weakly with DBP (*r* = −0.296, *p* = 0.035), Hb (*r* = −0.291, *p* = 0.042) and LDL-C (*r* = −0.334, *p* = 0.023), while sleep quality metrics calculated by CPC analysis were more significantly correlated with clinical indicators that were related to metabolic functions of T2DM patients. For example, liver function indicators AST/ALT was associated with TST (*r* = −0.575, *p* < 0.001) and UST (*r* = 0.424, *p* = 0.009), renal function indicator BUN was associated with TST (*r* = 0.488, *p* = 0.005), UST (*r* = 0.479, *p* = 0.006), ALUS (*r* = 0.442, *p* = 0.011) and USP (*r* = 0.392, *p* = 0.027). Moreover, TST and UST were significantly correlated with many clinical indicators, including admission FBG, Hb, ALT, AST/ALT, BUN, TG, HDL-C and LDL-C (e.g., TST & admission FBG, *r* = −0.383, *p* = 0.025; TST & TG, *r* = −0.405, *p* = 0.019; UST & LDL-C, *r* = −0.400, *p* = 0.017).

**TABLE 3:** Correlation coefficients between sleep quality metrics and clinical metabolic indicators.

Sleep Metabolic	PSQI	AHI	TST	UST	RST	ALUS	ALSS	ALRS	USP	SSP
Admission FBG			−0.383*^p^							
DBP	−0.296*^p^									
WBC							0.362*^s^			
Hb	−0.291*^p^	−0.337*^s^		−0.352*^p^		−0.385*^s^			−0.347*^p^	
ALT			−0.371*^s^	−0.334*^s^						
AST/ALT			0.575**^s^	0.424**^s^						
BUN			0.488**^s^	0.479**^s^		0.442*^s^			0.392*^s^	
UA								−0.375*^p^		
TG			−0.405*^s^							
HDL-C			0.386*^p^		0.348*^p^					
LDL-C	−0.334*^p^			−0.402*^p^					−0.363*^p^	0.357*^p^
UACR							0.423*^s^			

Values in the table indicate the level of correlation, *p < 0.05, ** *p* < 0.01. ^s^ represents Spearman correlation coefficient, and ^p^ represents Pearson correlation coefficient.

### 3.2 Significance of HRV metrics during sleep

Table 4 demonstrates the mean values of HRV metrics for 40 T2DM patients, which showed significant differences between sleep and awake periods, especially non-linear HRV metrics. During sleep period, subjects had smaller values of HR-mean, SDANN, TP, LF/HF and LFP, while their RMSSD, SDSD, SampEn, FuzzyEn, MSEsum5, MSEsum10, MSEsum20, MFEsum5, MFEsum10 and MFEsum20 were higher than those during awake period.

**TABLE 4:** HRV metrics for subjects during different recording period.

Metric (Unit)	24-h	Sleep	Awake	*p*-value
HR-mean (bpm)	78.99 ± 8.71	68.72 ± 8.24	83.57 ± 9.92	**<0.001†**
SDNN (ms)	114.66 (85.14, 147.21)	86.44 ± 29.33	90.41 ± 24.53	0.524
RMSSD (ms)	22.68 (14.97, 31.94)	23.87 (15.98, 39.39)	18.29 (14.37, 25.93)	**0.042†**
SDSD (ms)	22.68 (14.97, 31.94)	23.87 (15.98, 39.39)	18.29 (14.37, 25.93)	**0.042†**
SDANN (ms)	107.67 ± 34.20	57.51 (43.63, 71.61)	79.71 ± 23.38	**<0.001†**
pNN50 (%)	2.30 (0.63, 7.55)	3.59 (0.63, 17.81)	1.36 (0.56, 4.41)	0.061
TP (×10^7^ ms^2^)	4.49 ± 1.72	1.00 (0.71, 1.45)	1.38 (0.96, 1.87)	**0.038†**
VLF (×10^4^ ms^2^)	14.58 (9.02, 27.16)	6.19 (3.76, 9.68)	5.19 (2.44, 10.10)	0.473
LF (×10^4^ ms^2^)	3.92 (1.50, 6.97)	1.25 (0.61, 2.24)	1.51 (0.52, 2.71)	0.506
HF (×10^3^ ms^2^)	10.62 (5.56, 37.84)	4.50 (2.14, 15.49)	3.39 (1.38, 12.39)	0.383
LF/HF	2.58 (1.76, 3.29)	2.16 (1.39, 3.84)	3.73 ± 1.68	**0.006†**
LFP (%)	32.53 (32.07, 33.50)	32.46 (31.61, 33.17)	33.28 (32.58, 34.01)	**<0.001†**
HFP (%)	29.76 ± 1.55	29.53 ± 2.27	28.84 ± 1.42	0.117
DFA-α1	1.21 ± 0.18	1.20 ± 0.26	1.21 ± 0.17	0.848
DFA-α2	1.15 (1.10, 1.25)	1.20 ± 0.12	1.13 (1.06, 1.24)	0.071
ApEn	2.16 ± 0.31	2.21 (1.89, 2.31)	2.11 ± 0.31	0.771
SampEn	0.61 ± 0.23	1.14 ± 0.36	0.69 ± 0.23	**<0.001†**
FuzzyEn	1.41 ± 0.34	1.92 ± 0.47	1.43 ± 0.32	**<0.001†**
MSEsum5	3.43 ± 1.18	5.46 ± 1.55	3.99 ± 1.25	**<0.001†**
MSEsum10	7.75 ± 2.51	11.56 ± 3.00	9.08 ± 2.63	**<0.001†**
MSEsum20	16.88 ± 5.10	23.92 ± 5.75	19.95 ± 5.21	**0.002†**
MFEsum5	7.61 ± 1.85	9.55 ± 2.20	7.88 ± 1.83	**0.001†**
MFEsum10	16.52 ± 3.77	20.85 (18.06, 22.61)	17.23 ± 3.72	**0.007†**
MFEsum20	35.10 ± 7.25	42.22 (36.86, 46.05)	36.87 ± 7.06	**0.012†**

Data are presented as mean ± SD if the variable is normally distributed, otherwise presented as median (p25, p75). The bold font and † represent a significant difference between *Sleep* and *Awake*, i.e., *p* < 0.05.

Table 5 demonstrates correlation coefficients between HRV metrics in 24-h/sleep/awake periods and clinical indicators. During 24-h period, significant correlations could be observed between liver function indicators ALT, AST/ALT and HRV metrics (e.g., ALT & HF, *r* = 0.688, *p* < 0.001; AST/ALT & LF, *r* = −0.707, *p* < 0.001; AST/ALT & HF, *r* = −0.721, *p* < 0.001), and between renal function indicator BUN and HRV metrics (e.g., RMSSD, *r* = −0.482, *p* = 0.006; pNN50, *r* = −0.527, *p* = 0.002; HF, *r* = −0.517, *p* = 0.003); as to HRV metrics, HFP was significantly correlated with many clinical indicators (ALT, *r* = 0.466, *p* = 0.004; AST/ALT, *r* = −0.510, *p* = 0.001; BUN, *r* = −0.459, *p* = 0.007), and since HF component mainly reflects PNS activity (Pagani et al., 1986; Malliani et al., 1991; Montano et al., 2009), it might suggest that PNS function is closely linked to physiological status in T2DM patients. During sleep period, significant correlations could be observed between many HRV metrics and DBP (e.g., SampEn, *r* = 0.540, *p* < 0.001; MSE complexity, *r* = 0.571 ± 0.054, *p* < 0.001), Hb (e.g., MSE complexity, *r* = 0.513 ± 0.031, *p* = 0.001 ± 0.001; DFA-α2, *r* = −0.524, *p* < 0.001), ALT (e.g., HF, *r* = 0.638, *p* < 0.001; SampEn, *r* = 0.645, *p* < 0.001), AST/ALT (e.g., FuzzyEn, *r* = −0.693, *p* < 0.001; MFE complexity, *r* = −0.711 ± 0.021, *p* < 0.001), and BUN (e.g., pNN50, *r* = −0.556, *p* = 0.001; SampEn, *r* = −0.541, *p* = 0.002); meanwhile, the significant correlations were observed between many clinical indicators and non-linear HRV metrics (e.g., ALT & SampEn, *r* = 0.645, *p* < 0.001; ALT & FuzzyEn, *r* = 0.645, *p* < 0.001; AST/ALT & MFE complexity, *r* = −0.711 ± 0.021, *p* < 0.001). However, during awake period, the correlations between HRV metrics and clinical indicators decreased observably, and HRV metrics mainly correlated with liver function indicators ALT (e.g., LF, r = 0.632, *p* < 0.001; HF, *r* = 0.628, *p* < 0.001) and AST/ALT (e.g., LF, *r* = −0.592, *p* < 0.001; HF, *r* = −0.606, *p* < 0.001).

**TABLE 5:** Correlation coefficients between HRV metrics during different recording period and clinical indicators.

HRV Metabolic	RMSSD	pNN50	LF	HF	HFP	SampEn	MSE complexity	FuzzyEn	MFE complexity	DFA-α2
24-h										
Admission FBG			0.406*^s^							−0.507**^s^
Hb			0.471**^s^	0.412*^s^						−0.545**^s^
ALT	0.602**^s^	0.545**^s^	0.658**^s^	0.688**^s^	0.466**^s^					−0.519**^s^
AST			0.457**^s^	0.488**^s^						−0.436**^s^
AST/ALT	−0.572**^s^	−0.650**^s^	−0.707**^s^	−0.721**^s^	−0.510**^s^					0.531**^s^
BUN	−0.482**^s^	−0.527**^s^	−0.465**^s^	−0.517**^s^	−0.459**^p^					0.430**^s^
UACR						−0.458*^s^	−0.492 ± 0.005**^s^	−0.361*^s^	−0.478 ± 0.008**^s^	
Sleep										
Admission FBG							0.413 ± 0.034*^p^		0.414 ± 0.010*^s^	−0.575**^p^
DBP			0.414**^s^	0.483**^s^		0.540**^p^	0.571 ± 0.054**^p^	0.528**^p^	0.537 ± 0.015**^s^	−0.458**^p^
Hb	0.430**^s^	0.418**^s^	0.505**^s^	0.430**^s^		0.491**^p^	0.513 ± 0.031**^p^	0.495**^p^	0.472**^s^	−0.524**^p^
ALT	0.629**^s^	0.573**^s^	0.624**^s^	0.638**^s^	0.449**^s^	0.645**^s^	0.574 ± 0.032**^s^	0.645**^s^	0.619 ± 0.028**^s^	−0.435**^s^
AST	0.440**^s^		0.449**^s^	0.434**^s^		0.487**^s^	0.449 ± 0.019**^s^	0.449**^s^	0.447 ± 0.001*^s^	
AST/ALT	−0.692**^s^	−0.657**^s^	−0.660**^s^	−0.691**^s^	−0.496**^s^	−0.641**^s^	−0.593 ± 0.045**^s^	−0.693**^s^	−0.711 ± 0.021**^s^	0.500**^s^
GGT						0.489**^s^	0.492 ± 0.011**^s^	0.407*^s^	0.454 ± 0.021**^s^	
BUN	−0.535**^s^	−0.556**^s^	−0.436*^s^	−0.522**^s^	−0.409*^p^	−0.541**^p^	−0.525**^p^	−0.521**^p^	−0.533 ± 0.041**^s^	
UACR							−0.400 ± 0.013**^s^	−0.512**^s^		
Awake										
Hb			0.438**^s^							−0.662**^s^
ALT	0.522**^s^	0.498**^s^	0.632**^s^	0.628**^s^	0.523**^s^					−0.540**^s^
AST			0.486**^s^	0.461**^s^						−0.439*^s^
AST/ALT	−0.579**^s^	−0.583**^s^	−0.592**^s^	−0.606**^s^	−0.539**^s^					0.554**^s^
BUN	−0.441*^s^	−0.491**^s^	−0.455*^s^	−0.466**^s^	−0.431*^p^		−0.403 ± 0.028*^p^		−0.404 ± 0.029*^s^	0.496**^s^

Values in the table indicate the level of correlation, *p < 0.05, ** *p* < 0.01. ^s^ represents Spearman correlation coefficient, and ^p^ represents Pearson correlation coefficient. Only metrics or indicators that had several significant correlations with others are selected, and only correlation coefficients whose absolute value were higher than 0.4 are presented here, while others are presented in Supplementary Table S1.

It could be seen that the overall level of correlation coefficients was significantly higher during sleep period than 24-h and awake periods. Correlations between variability-related HRV metrics (RMSSD and pNN50) and clinical indicators, including liver function (ALT, AST/ALT) and renal function (BUN), were more significant in sleep period than in 24-h and in awake period; significant correlations between complexity-related HRV metrics (SampEn, FuzzyEn, MSE complexity, MFE complexity) and clinical indicators were found mainly in sleep period.

### 3.3 HRV metrics in different sleep stages are more associated with T2DM clinical indicators

To investigate the relationship between HRV metrics (ANS function) in different sleep stages and the clinical indicators (metabolic function) of T2DM patients, the RR interval time series during sleep period were segmented according to sleep stages, including unstable sleep, stable sleep and REM sleep, and series from the same sleep stage were stitched together as a whole. Significant differences were observed among HRV metrics in different sleep stages. For example, the LF/HF reached its maxima in unstable sleep (4.04 ± 3.31), followed by REM sleep (3.36 ± 2.27) and its minima in stable sleep (1.45 ± 0.98). More details are shown in Supplementary Table S2.

The correlation coefficients between HRV metrics in different sleep stages and clinical indicators are demonstrated in Table 6. Linear HRV metrics, especially RMSSD, SDSD, pNN50, LF, HF and HFP were correlated significantly with Hb, ALT, AST/ALT and BUN (e.g., in unstable sleep: SDSD & ALT, *r* = 0.665, *p* < 0.001; RMSSD & AST/ALT, *r* = −0.710, *p* < 0.001; HF & AST/ALT, *r* = −0.716, *p* < 0.001; pNN50 & BUN, *r* = −0.533, *p* = 0.002; in stable sleep: HFP & AST/ALT, *r* = −0.556, *p* < 0.001; in REM sleep: LF & Hb, *r* = 0.510, *p* = 0.001). In non-linear HRV analysis, more metrics were tightly associated with clinical indicators, and the correlations were more significant with those in linear analysis (63 correlations with |r|>0.5, compared with 44 correlations with |r|>0.5 in linear analysis), especially the DFA metric α2 and the complexity metrics MSE and MFE (e.g., in unstable sleep: DFA-α2 & admission FBG, *r* = −0.584, *p* < 0.001; MFE & AST/ALT, r = −0.614 ± 0.052, *p* < 0.001; MFE complexity & AST/ALT, *r* = −0.680 ± 0.014, *p* < 0.001; in stable sleep: SampEn & ALT, *r* = 0.786, *p* < 0.001; MSE complexity & ALT, *r* = 0.596 ± 0.076, *p* < 0.001; MSE & AST/ALT, *r* = −0.571 ± 0.132, *p* = 0.002 ± 0.003; FuzzyEn & AST/ALT, *r* = −0.760, *p* < 0.001). Compared with the correlation with linear HRV metrics, correlations between admission FBG, DBP, Hb, ALT, AST, AST/ALT, GGT, BUN and non-linear HRV metrics increased considerably (e.g., in unstable sleep: DFA-α2 & admission FBG, *r* = −0.584, *p* < 0.001; in stable sleep: SampEn & ALT, *r* = 0.786, *p* < 0.001; SampEn & AST, *r* = 0.630, *p* < 0.001; FuzzyEn & AST/ALT, *r* = −0.760, *p* < 0.001; SampEn & BUN, *r* = −0.625, *p* < 0.001; in REM sleep: MSE complexity & DBP, *r* = 0.560 ± 0.046, *p* < 0.001; DFA-α2 & Hb, *r* = −0.549, *p* < 0.001; MSE complexity & GGT, *r* = 0.494 ± 0.009, *p* = 0.002 ± 0.000), and significant association between HbA1c, N%, PLT, HDL-C, LDL-C, UMA and HRV metrics were mainly observed in non-linear analysis (e.g., in unstable sleep: FuzzyEn & HbA1c, *r* = 0.506, *p* = 0.001; in stable sleep: MFE & N%, *r* = −0.474, *p* = 0.003; in REM sleep: MFE & PLT, *r* = 0.515, *p* = 0.001; DFA-α2 & LDL-C, *r* = −0.495, *p* = 0.003; MFE & UMA, *r* = −0.512 ± 0.008, *p* = 0.003 ± 0.001).

**TABLE 6:** Correlation coefficients between HRV metrics in three sleep stages and clinical indicators.

(a) Correlation coefficients between linear HRV metrics and clinical indicators							
Linear HRV Metabolic	Time-domain			Frequency-domain			
	RMSSD	SDSD	pNN50	VLF	LF	HF	HFP
Unstable sleep							
Hb	0.451**^s^	0.451**^s^	0.406*^s^		0.472**^s^	0.433**^s^	
ALT	0.665**^s^	0.665**^s^	0.610**^s^	0.436**^s^	0.525**^s^	0.682**^s^	0.519**^s^
AST	0.477**^s^	0.477**^s^	0.423*^s^		0.410*^s^	0.508**^s^	
AST/ALT	−0.710**^s^	−0.710**^s^	−0.701**^s^	−0.416*^s^	−0.540**^s^	−0.716**^s^	−0.551**^s^
BUN	−0.516**^s^	−0.516**^s^	−0.533**^s^			−0.476**^s^	
Stable Sleep							
ALT	0.568**^s^	0.568**^s^	0.525**^s^		0.510**^s^	0.581**^s^	0.489**^s^
AST	0.403*^s^	0.403*^s^				0.410*^s^	
AST/ALT	−0.654**^s^	−0.654**^s^	−0.620**^s^	−0.428**^s^	−0.554**^s^	−0.623**^s^	−0.556**^s^
BUN	−0.506**^s^	−0.506**^s^	−0.493**^s^		−0.478**^s^	−0.524**^s^	−0.414*^s^
REM Sleep							
Hb	0.410*^s^	0.410*^s^			0.510**^s^	0.478*^s^	
ALT	0.586**^s^	0.586**^s^	0.552**^s^		0.475**^s^	0.490*^s^	0.430**^s^
AST/ALT	−0.680**^s^	−0.680**^s^	−0.699**^s^	−0.405*^s^	−0.489**^s^	−0.561**^s^	−0.509**^s^
BUN	−0.507**^s^	−0.507**^s^	−0.529**^s^		−0.410*^s^	−0.524**^s^	−0.483**^p^

(b) Correlation coefficients between non-linear HRV metrics and clinical indicators							
Non-linear HRV Metabolic	DFA-α2	SampEn	MSE	MSE complexity	FuzzyEn	MFE	MFE complexity
Unstable Sleep							
Admission FBG	−0.584**^p^	0.400*^p^	0.466**^s^	0.440**^p^		0.436*^s^	0.403 ± 0.003*^p^
HbA1c	−0.412*^p^	0.438*^p^	0.411 ± 0.016*^s^	0.409 ± 0.017*^p^			
DBP		0.523**^p^	0.488 ± 0.047**^s^	0.545 ± 0.060**^p^		0.457 ± 0.043**^s^	0.543 ± 0.039**^p^
Hb	−0.496**^p^	0.483**^p^	0.424 ± 0.001**^s^	0.494 ± 0.031**^p^	0.500**^p^	0.435 ± 0.009**^s^	0.490 ± 0.051**^p^
ALT		0.572**^s^	0.472 ± 0.047**^s^	0.520 ± 0.029**^p^	0.615**^s^	0.538 ± 0.048**^s^	0.590 ± 0.012**^s^
AST			0.426**^s^		0.440**^s^	0.461 ± 0.043**^s^	0.439**^s^
AST/ALT	0.421*^s^	−0.614**^s^	−0.503 ± 0.063**^s^	−0.583 ± 0.040**^p^	−0.656**^s^	−0.614 ± 0.052**^s^	−0.680 ± 0.014**^s^
GGT			0.455 ± 0.034**^s^	0.452**^p^		0.478 ± 0.048**^s^	0.459**^s^
BUN		−0.443*^p^	−0.400 ± 0.035*^s^	−0.408 ± 0.044*^p^	−0.451*^p^	−0.488 ± 0.032**^s^	−0.463**^p^
LDL-C		0.460**^p^			0.428*^s^		
Stable Sleep							
DBP		0.458**^s^	0.463 ± 0.039**^s^	0.447**^s^	0.508**^p^	0.456 ± 0.033**^s^	0.474 ± 0.027**^s^
N%			−0.462 ± 0.031**^s^	−0.461 ± 0.035*^s^		−0.474**^s^	
Hb	−0.535**^p^	0.498**^s^	0.469 ± 0.037**^s^	0.476 ± 0.070*^s^	0.475**^p^		0.431**^s^
ALT	−0.419*^s^	0.786**^s^	0.553 ± 0.114**^s^	0.596 ± 0.076**^s^	0.721**^s^	0.580 ± 0.070**^s^	0.635 ± 0.042**^s^
AST	−0.496**^s^	0.630**^s^	0.523 ± 0.058**^s^	0.555 ± 0.051**^s^	0.522**^s^	0.488 ± 0.054**^s^	0.491 ± 0.035**^s^
AST/ALT	0.376*^s^	−0.728**^s^	−0.571 ± 0.132**^s^	−0.516 ± 0.095**^s^	−0.760**^s^	−0.585 ± 0.075**^s^	−0.667 ± 0.055**^s^
GGT		0.441**^s^	0.434 ± 0.011**^s^		0.442*^s^	0.482 ± 0.060**^s^	0.447 ± 0.007*^s^
BUN		−0.625*^s^	−0.570 ± 0.078**^s^		−0.576**^p^	−0.545 ± 0.072**^s^	−0.534 ± 0.047**^s^
REM Sleep							
Admission FBG	−0.524**^p^		0.403 ± 0.023*^s^				
HbA1c		0.424*^p^	0.418 ± 0.022*^s^	0.442 ± 0.021*^p^			
DBP	−0.507**^p^	0.493**^p^	0.502 ± 0.070**^s^	0.560 ± 0.046**^p^	0.496**^p^	0.489 ± 0.049**^s^	0.533 ± 0.029**^s^
Hb	−0.549**^p^	0.475**^p^	0.479 ± 0.037**^s^	0.524 ± 0.043**^p^	0.504**^p^	0.478 ± 0.054**^s^	0.464 ± 0.040**^s^
PLT						0.515**^s^	
ALT	−0.402*^s^	0.581**^s^	0.512 ± 0.041**^s^	0.552 ± 0.011**^s^	0.591**^s^	0.543 ± 0.061**^s^	0.590 ± 0.007**^s^
AST		0.416*^s^	0.437 ± 0.019**^s^	0.432 ± 0.003*^s^	0.416*^s^	0.476 ± 0.039**^s^	0.402 ± 0.008*^s^
AST/ALT	0.437**^s^	−0.575**^s^	−0.493 ± 0.042**^s^	−0.518 ± 0.029**^s^	−0.627**^s^	−0.570 ± 0.070**^s^	−0.649 ± 0.001**^s^
GGT		0.478**^s^	0.462 ± 0.023**^s^	0.494 ± 0.009**^s^	0.400*^s^	0.468 ± 0.026**^s^	0.436**^s^
BUN		−0.472**^p^	−0.495 ± 0.030**^s^	−0.486**^p^	−0.496**^p^	−0.535 ± 0.043**^s^	−0.578 ± 0.010**^s^
LDL-C	−0.495**^p^	0.422*^p^		0.400 ± 0.010*^p^	0.400*^p^	0.484 ± 0.036**^s^	
UMA			−0.466*^s^			−0.512 ± 0.008**^s^	
UACR			−0.505 ± 0.027**^s^	−0.488 ± 0.004**^s^		−0.514 ± 0.046**^s^	−0.404 ± 0.003*^s^

Values in the table indicate the level of correlation, *p < 0.05, ** *p* < 0.01. ^s^ represents Spearman correlation coefficient, and ^p^ represents Pearson correlation coefficient. Only metrics or indicators that had several significant correlations with others are selected, and only correlation coefficients whose absolute value were higher than 0.4 are presented here, while others are presented in Supplementary Table S3.

The findings hinted that HRV metrics in different sleep stages were more associated with T2DM clinical indicators. During each sleep stage, the correlation coefficients between HRV metrics and clinical indicators demonstrated a higher overall level compared with those during sleep period. Compared with linear HRV metrics, non-linear HRV metrics significantly correlated with more clinical indicators, including LDL-C and UACR.

### 3.4 Non-linear HRV metrics during sleep are associated with glycemic control

Interestingly, non-linear HRV metrics in different sleep stages were significantly associated with glycemic control, while few weak correlations were found between HRV metrics of 24-h and awake periods and glycemic control indicators. Table 7 demonstrates correlation coefficients between non-linear HRV metrics in three sleep stages and glycemic control indicators including admission FBG, discharge FBG and HbA1c. Most of non-linear HRV metrics were significantly correlated with admission FBG (e.g., in unstable sleep: DFA-α2, *r* = −0.584, *p* < 0.001; MSE, *r* = 0.466, *p* = 0.006; MSE complexity, *r* = 0.440, *p* = 0.009), yet had no or only weak correlation with discharge FBG. Both single- and multiple-scale sample entropies showed significant correlations with HbA1c (in unstable sleep: SampEn, *r* = 0.438, *p* = 0.025; MSE, *r* = 0.411 ± 0.016, *p* = 0.038 ± 0.008; MSE complexity, *r* = 0.409 ± 0.017, *p* = 0.039 ± 0.009; in stable sleep: MSE, *r* = 0.389, *p* = 0.050; in REM sleep: SampEn, *r* = 0.424, *p* = 0.031; MSE, *r* = 0.418 ± 0.022, *p* = 0.035 ± 0.010; MSE complexity, *r* = 0.442 ± 0.021, *p* = 0.024 ± 0.007).

**TABLE 7:** Correlation coefficients between non-linear HRV metrics in three sleep stages and clinical glycemic control indicators including admission FBG, discharge FBG and HbA1c.

Non-linear HRV Glycemic control	DFA-α2	SampEn	MSE	MSE complexity	FuzzyEn	MFE	MFE complexity
Unstable Sleep							
Admission FBG	−0.584**^p^	0.400*^p^	0.466**^s^	0.440**^p^	0.378*^p^	0.436*^s^	0.403 ± 0.003*^p^
HbA1c	−0.412*^p^	0.438*^p^	0.411 ± 0.016*^s^	0.409 ± 0.017*^p^			
Stable Sleep							
Admission FBG		0.362*^s^	0.353 ± 0.015*^s^	0.364*^s^		0.377*^s^	
Discharge FBG			−0.423*^s^				
HbA1c			0.389*^s^				
REM Sleep							
Admission FBG	−0.524**^p^		0.403 ± 0.023*^s^			0.394 ± 0.024*^s^	
HbA1c		0.424*^p^	0.418 ± 0.022*^s^	0.442 ± 0.021*^p^			

Values in the table indicate the level of correlation, *p < 0.05, ** *p* < 0.01. ^s^ represents Spearman correlation coefficient, and ^p^ represents Pearson correlation coefficient.

Furthermore, the subjects were divided into two groups according to whether the subject had poor control of diabetes (HbA1c ≥ 9.0%, *n* = 10) or not (HbA1c < 9.0%, *n* = 16). Table 8 demonstrates between-group differences in non-linear HRV metrics. Significant differences were observed in non-linear metrics in unstable and stable sleep (in unstable sleep: DFA-alpha2, *p* = 0.002; MSEsum5, *p* = 0.020; MSEsum10, *p* = 0.017; MSEsum20, *p* = 0.037; in stable sleep: SampEn, *p* = 0.020; DFA-alpha2, *p* = 0.026; MSEsum5, *p* = 0.002; MSEsum10, *p* = 0.004; MSEsum20, *p* = 0.024).

**TABLE 8:** Difference in non-linear HRV metrics between the group had poor control of diabetes (HbA1c ≥ 9.0%) or did not (HbA1c < 9.0%). Data are presented as mean ± SD.

Sleep Stage	Non-linear HRV metric	HbA1c < 9.0% (n = 16)	HbA1c ≥ 9.0% (n = 10)	*p*-value
Unstable Sleep	DFA-α2	1.28 ± 0.09	1.15 ± 0.09	0.002
	MSEsum5	5.07 ± 1.20	6.19 ± 0.98	0.020
	MSEsum10	11.24 ± 2.47	13.63 ± 2.04	0.017
	MSEsum20	24.79 ± 5.03	29.03 ± 4.32	0.037
Stable Sleep	SampEn	1.42 ± 0.31	1.69 ± 0.18	0.020
	DFA-α2	1.11 ± 0.15	0.99 ± 0.08	0.026
	MSEsum5	6.54 ± 1.59	8.14 ± 0.69	0.002
	MSEsum10	13.33 ± 3.12	16.26 ± 1.48	0.004
	MSEsum20	26.31 ± 6.02	31.29 ± 3.12	0.024

### 3.5 Association among glycemic control, non-linear HRV analysis and sleep

We also applied partial correlation analysis between HbA1c, admission FBG and non-linear HRV metrics in sleep stages as controlling the effect of sleep quality metrics, in order to briefly discover the interaction among glycemic control, non-linear HRV analysis and sleep.

Table 9 demonstrates partial correlations between HbA1c and non-linear HRV metrics in unstable and REM sleep. When nullifying the effect of SST, ALSS and SSP, the correlations between HbA1c and non-linear HRV metrics significantly strengthened (e.g., SampEn in REM sleep: zero order, *r* = 0.424, *p* = 0.031; nullified by SST, *r* = 0.547, *p* = 0.005; nullified by ALSS, *r* = 0.549, *p* = 0.004; nullified by SSP, *r* = 0.569, *p* = 0.003). However, the correlations between HbA1c and non-linear HRV metrics decreased and even were lost when corrected for TST (e.g., SampEn in REM sleep: zero order, *r* = 0.424, *p* = 0.031; nullified by TST, *r* = 0.402, *p* = 0.046; MSEsum10 in REM sleep: zero order, *r* = 0.428, *p* = 0.029; nullified by TST, *r* = 0.400, *p* = 0.048). The associations were mostly retained when corrected for other sleep metrics (e.g., MSEsum5 in unstable sleep: zero order, *r* = 0.421, *p* = 0.032; nullified by AHI, *r* = 0.428, *p* = 0.033; nullified by ALUS, *r* = 0.417, *p* = 0.038; nullified by USP, *r* = 0.424, *p* = 0.035).

**TABLE 9:** Partial correlation analysis between HbA1c and non-linear HRV metrics in unstable and REM sleep.

Glycemic control Non-linear HRV		HbA1c								
		Zero order	Nullified by AHI	Nullified by TST	Nullified by SST	Nullified by RST	Nullified by ALUS	Nullified by ALSS	Nullified by USP	Nullified by SSP
Unstable Sleep	SampEn	0.438*	0.474*		0.489*	0.453*	0.442*	0.527**	0.465*	0.532**
	MSEsum5	0.421*	0.428*		0.445*	0.457*	0.417*	0.469*	0.424*	0.468*
	MSEsum10	0.397*	0.396*		0.405*	0.458*	0.398*	0.422*		0.421*
REM Sleep	SampEn	0.424*	0.510**	0.402*	0.547**	0.411*	0.452*	0.549**	0.495*	0.569**
	MSEsum5	0.457*	0.505**	0.431*	0.552**	0.455*	0.470*	0.561**	0.499*	0.573**
	MSEsum10	0.428*	0.456*	0.400*	0.506**	0.438*	0.430*	0.534**	0.450*	0.524**

Values in the table indicate the level of correlation, *p < 0.05, ** *p* < 0.01.

Table 10 demonstrates partial correlations between admission FBG and non-linear HRV metrics in 3 sleep stages. When nullifying the effect of SST, the correlations between HbA1c and non-linear HRV metrics at sleep stages significantly strengthened (e.g., SampEn in unstable sleep: zero order, *r* = 0.400, *p* = 0.019; nullified by SST, *r* = 0.464, *p* = 0.007; FuzzyEn in unstable sleep: zero order, *r* = 0.378, *p* = 0.027; nullified by SST, r = 0.466, *p* = 0.006). When nullifying the effect of TST, the correlations between HbA1c and non-linear HRV metrics decreased and even were lost (e.g., MSEsum5 in unstable sleep: zero order, *r* = 0.440, *p* = 0.009; nullified by TST, *r* = 0.346, *p* = 0.049; DFA-α2 in REM sleep: zero order, *r* = −0.524, *p* = 0.001; nullified by TST, *r* = −0.465, *p* = 0.006). The associations between admission FBG and non-linear HRV metrics, especially DFA-α2, were retained when corrected for other sleep metrics (e.g., DFA-α2 in unstable sleep: zero order, *r* = −0.584, *p* < 0.001; nullified by AHI, r = −0.585, *p* < 0.001; nullified by USP, *r* = −0.582, *p* < 0.001; nullified by SSP, *r* = −0.586, *p* < 0.001; DFA-α2 in REM sleep: zero order, *r* = −0.524, *p* = 0.001; nullified by RST, *r* = −0.537, *p* = 0.001; nullified by ALUS, *r* = −0.538, *p* = 0.001).

**TABLE 10:** Partial correlation analysis between admission FBG and non-linear HRV metrics in 3 sleep stages.

Glycemic control Non-linear HRV		Admission FBG								
		Zero order	Nullified by AHI	Nullified by TST	Nullified by UST	Nullified by SST	Nullified by RST	Nullified by ALUS	Nullified by USP	Nullified by SSP
Unstable Sleep	DFA-α2	−0.584**	−0.585**	−0.549**	−0.569**	−0.595**	−0.580*	−0.594**	−0.582**	−0.586**
	SampEn	0.400*	0.435*		0.345*	0.464**	0.396*	0.398*	0.416*	0.437*
	MSEsum5	0.440**	0.448**	0.346*	0.397*	0.473**	0.433*	0.435*	0.440**	0.455**
	MSEsum10	0.425*	0.425*		0.393*	0.437*	0.419*	0.426**	0.422*	0.429*
	FuzzyEn	0.378*	0.413*			0.466**	0.375*	0.375*	0.403*	0.430*
	MFEsum5	0.405*	0.419*		0.353*	0.467**	0.399*	0.399*	0.413*	0.435*
	MFEsum10	0.401*	0.405*		0.353*	0.444**	0.393*	0.397*	0.400*	0.418*
Stable Sleep	MSEsum5	0.354*	0.353*			0.369*	0.354*	0.349*	0.350*	0.354*
REM Sleep	DFA-α2	−0.524**	−0.561**	−0.465**	−0.496**	−0.615**	−0.537**	−0.538**	−0.571**	−0.578**

Values in the table indicate the level of correlation, *p < 0.05, ** *p* < 0.01.

## 4 Discussion

This study focused on the relationships among sleep, HRV derived ANS function and metabolic function in T2DM, and the main findings were: 1) metabolic function was significantly correlated with sleep quality; 2) HRV derived ANS function was different between sleep and awake, showing strengthened and distinct distributions of correlations with metabolic function compared to 24-h analysis; 3) HRV derived ANS function and its correlation with clinical indicators of metabolic function altered during sleep cycles; 4) HRV analysis during sleep was strongly associated with clinical indicators of metabolic function, and this also applied to the glycemic control in patients with T2DM in non-linear analysis. These findings demonstrated that sleep rhythm based HRV analysis should be emphasized in ANS and metabolic function assessment and the rationality of non-linear HRV analysis for further application among T2DM patients and investigation of potential interactions among ANS, sleep and metabolic function (Lombardi, 2011; Perkiömäki, 2011).

In sleep quality assessment, PSQI had few and weak correlations with clinical indicators, while TST and UST correlated with more clinical indicators. The results suggest limitations of PSQI from its objectivity and indicate the important role of sleep in regulation of metabolic function. Researchers have found that circadian rhythms and sleep regulate hormones and lipids involved in energy metabolism (VanCauter et al., 1997; Scheer et al., 2009; Nguyen and Wright, 2010; Spiegel et al., 2011; Markwald et al., 2013), and disruption of sleep and circadian rhythms is progressively apparent as an influence factor to damaged physiological function, disease processes and metabolic dysregulation (Spiegel et al., 2005; Turek et al., 2005; Marcheva et al., 2010; Markwald and Wright, 2012).

The enhanced association between ANS and metabolic function during sleep is consistent with previously published results. Researchers have found that most of the biological functions changes during sleep compared to wake, such as heart rate, arterial blood pressure and etc., where ANS plays a vital role (Tobaldini et al., 2017). Results in Table 5, 6 showed that HRV metrics during sleep were associated with clinical indicators of metabolic function more significantly and broadly, compared to 24-h HRV, and there were significant differences between HRV metrics during sleep and awake. Thus, sleep might be a good model to investigate ANS activity and the fluctuation of ANS function caused by intrinsic factors, especially circadian rhythm, without influence factors of daytime activities such as eating behaviour, physical movement, etc. Meanwhile, studies have shown that HRV metrics are significantly influenced by routine life states and ECG recording time (morning, noon, evening), and the RR values are rather stable during night sleeping (Cui et al., 2020), so we analysed the RR interval time series during awake period (i.e., during daytime) as a whole, and at the same time the time of waking and sleeping of subjects was rather uniform, which may decrease bias brought by recording time.

Significant differences observed in many HRV metrics among different sleep stages suggest the dominance of SNS in unstable sleep and the dominance of PNS in stable sleep (Pagani et al., 1986; Malliani et al., 1991; Montano et al., 2009). Researchers have found that ANS tone and modulation are profoundly influenced by sleep related mechanisms (Somers et al., 1993; Trinder et al., 2001; Tobaldini et al., 2014; de Zambotti et al., 2018), while cardiovascular function within each sleep stage tends not to vary across the night (Silvani and Dampney, 2013). For ANS impose regulatory control over the cardiovascular system by SNS and PNS, which leads to HRV, the changes of HRV metrics in different sleep stages and their correlations with clinical indicators may associate more closely with the variability of ANS function during sleep cycles. Researchers have conducted HRV analysis during sleep to confirm different ANS function patterns at sleep stages (Scholz et al., 1997; Brandenberger et al., 2001; Trinder et al., 2001; Stein and Pu, 2012). Studies have shown that, since most of HRV metrics applied in this study were based on the assumption of stationary series (Akselrod et al., 1981; Pagani et al., 1986; Pincus et al., 1993; Task Force of the European Society of Cardiology the North American Society of Pacing Electrophysiology, 1996; Voss et al., 1996; Cooke et al., 1999; Porta et al., 2000b; Cysarz et al., 2000; Richman and Moorman, 2000; Wessel et al., 2000; Porta et al., 2001), non-stationarities of HRV sequences could have a great influence on the assessment of the balance of ANS by producing a bias toward a higher sympathetic modulation and a lower vagal modulation (Magagnin et al., 2011). We applied Augmented Dickey-Fuller test to all the HRV sequences for stationarity test, and found sequences during 24-h, daytime and sleep were mostly stationary. However, during sleep cycles, the sequences were mostly non-stationary. Hence, the distinct distribution pattern presented in analysis during sleep cycles may demonstrate the shift of sympathovagal balance toward sympathetic predominance since non-stationarities of the HRV sequences will highlight the shift (Magagnin et al., 2011). With enhanced and clarified interaction between ANS function and HRV analysis in each sleep stage, the underlying autonomic pathways (Stein and Pu, 2012) of different HRV metrics may be better distinguished from the remarkably strengthened correlations between clinical indicator of metabolic function and HRV metrics with distinct distribution patterns compared to traditional analysis of sleep quality and HRV, which is crucial to reveal the interaction among sleep, ANS and HRV.

Compared to linear analysis, non-linear HRV metrics in different sleep stages were associated with more T2DM clinical indicators. According to the development of chaotic theory and non-linear dynamics, it is now generally recognized that heart beat intervals (RR intervals) are non-linear and non-stationary time series, caused by complex interactions between physiological systems (Peng et al., 1995; Cerutti et al., 2007). Traditional methods in time- and frequency-domain may not be able to detect subtle but important changes in RR series (Costa et al., 2005). Currently, HRV frequency-domain metrics are extensively used to assess the function of ANS components, leading to controversial results (Stein and Pu, 2012; Reyes del Paso et al., 2013; Martelli et al., 2014). In this study, most comprehensive and strong correlations between HRV derived ANS function and metabolic function with distinct distribution patterns were observed in non-linear analysis, demonstrating the rationality of non-linear analysis in assessing ANS function.

However, a part of non-linear HRV metrics we chose are somehow influenced by some biases. ApEn is a biased statistic, and the bias arises from two separate sources. First, in the calculation of the correlation integral $C_{i}^{m}\left(r\right)$ , the vector $X\left(i\right)$ counts itself to ensure that the logarithms remain finite, which is defined as “self-matching,” underestimating the calculation of conditional probabilities as a consequence of it and producing a bias towards regularity. If the number of matches between templates is low, the bias can be as high as 20% or 30%. Second, the concavity of the logarithm implies a bias in all the regularity statistics (Pincus and Huang, 1992; Delgado-Bonal and Marshak, 2019). On some occasions, ApEn failed to capture the sympathovagal balance (Porta et al., 2007a). The MSE method also has shortcomings. The course of the entropy-based complexity as a function of the time scale is partially linked to the reduction of variance inherent to the procedure for the elimination of the fast temporal scales, which confines more and more patterns inside phase space cells of constant dimension (Nikulin and Brismar, 2004). As a consequence, more and more patterns become indistinguishable, thus artificially reducing complexity with the scale factor τ. Obviously, this effect is more evident at large time scales when variance is importantly reduced, thus producing biased MSE courses especially at large time scales. Meanwhile, the procedure devised to eliminate fast time scales is suboptimal, thus producing uncontrolled effects on the assessment of complexity at any scale (Valencia et al., 2009).

It is likewise not negligible that the impact of the data length on the ability to predict the health status of ANS (Laborde et al., 2017). Long-term 24-h HRV recordings are acknowledged as the “gold standard” for clinical HRV assessment (Shaffer et al., 2014) and achieve greater predictive power than short-term measurements (Bigger et al., 1989; Fei et al., 1996; Nolan et al., 1998; Kleiger et al., 2005). However, increased HRV may correspond to longer recordings instead of the change of ANS function, and it is inappropriate to compare metrics like SDNN when they are calculated from epochs of different length (Saul et al., 1988; Task Force of the European Society of Cardiology the North American Society of Pacing Electrophysiology, 1996). Meanwhile, since mechanisms responsible for heart period modulations of a certain frequency, especially LF and HF power components, may not remain unchanged during long-term recording (Furlan et al., 1990), the interpretation of the results of frequency analysis is less well defined, and they may obscure the detailed information about autonomic modulation of RR intervals that is available in shorter recordings (Task Force of the European Society of Cardiology the North American Society of Pacing Electrophysiology, 1996).

In this study, surrogate tests (Porta et al., 2000a) was applied to all the HRV sequences analysed. The percentages of non-linear dynamics were: 1) 86.05% in 24-h HRV sequences; 2) 90.70% during awake period, and 39.53% during sleep period; 3) 52.63% during stable sleep, 55.26% during unstable sleep, and 68.42% during REM sleep. Therefore, non-linear dynamics in HRV sequences decreased during sleep, possibly due to the suppression of regulation of respiratory rhythm (Mador and Tobin, 1991; González et al., 2000; Francis et al., 2002; Jo et al., 2005), leading to stable patterns. And when HRV sequences were segmented based on sleep stages, the percentage of non-linear dynamics elevated, suggesting better manifestation of the non-linear components in HRV sequences during sleep cycles and demonstrating the effectiveness of non-linear analysis. Additionally, the distribution of non-linear features during different sleep stages, that the maximum of non-linear dynamics appeared during REM sleep, was consistent with non-linear respiratory dynamics (Sako et al., 2001).

However, researchers have found that in the analysis of short-term, stationary and successive HRV sequences, non-linear model-free and linear model-based conditional entropy correlate closely and have similar performance in the assessment of cardiac control (Porta et al., 2017). The conflicting conclusions may be a consequence of differences in stationary and non-linear features of HRV sequences utilized in analysis (Magagnin et al., 2011). Previous study has shown that short-term HRV in healthy young adults at rest is mainly linear (Porta et al., 2007b), while in this study HRV sequences during sleep cycles were mainly non-stationary and had non-linear features, demonstrating that the performance of non-linear analysis is related to the feature of HRV sequences.

Sleep rhythm based HRV analysis presents us significant correlations between ANS and metabolic function of T2DM patients covered by limitations of traditional measures, demonstrating its efficiency on distinguishing potential dynamic characteristics of ANS function and its correlation with metabolic function in different sleep stages. Hence, by analysing the correlation between ANS function patterns and metabolic function of T2DM patients, our findings propose a promising strategy for the evaluation of T2DM patients’ metabolic function and physical condition to improve the early prevention, post-diagnosis and management of T2DM and its complications based on HRV analysis during sleep cycles.

Significant correlations were found among DBP and HRV metrics during sleep (see Table 5), and non-linear HRV metrics during sleep cycles significantly correlated with DBP with distinct distribution patterns (see Table 6), suggesting strong association between cardiac baroreflex control and the fluctuation of HRV metrics, especially non-linear ones, in T2DM patients. According to the SBP (136 ± 14 mmHg) and DBP (85 ± 11 mmHg) of the study cohort shown in Table 1 and “*Clinical practice guidelines for the management of hypertension in China*” (National Center for Cardiovascular Diseases et al., 2022), plenty of the subjects had hypertension of varying degrees, suggesting impairment of cardiac baroreflex control, associated with impairment of ANS and the presence of other neuropathies (Frattola et al., 1997; Tank et al., 2001; Ruiz et al., 2005; Vinik and Ziegler, 2007), which may be the effect of T2DM on baroreflex control through an impairment of vagal control (de Moura-Tonello et al., 2016), and the significant correlations support the finding that cardiovascular variability indexes are sensitive to autonomic dysfunction in T2DM patients in early stages (de Moura-Tonello et al., 2016). These findings demonstrate the effectiveness of non-linear HRV analysis and promising strategies for the early detection of autonomic dysfunction.

ANS plays an important role in the exchange of metabolic information between organs and regulation on peripheral metabolism (Yamada and Katagiri, 2007). In addition to affecting the activity of ANS, sleep also exerts a significant regulatory effect on glycemic control by influencing the balance and levels of hormones including leptin, growth hormone-releasing peptide, insulin and cortisol (Balbo et al., 2010), which enhances the association between HRV metrics during sleep and glycemic control of T2DM patients. Researchers have found that 24-h HRV metrics in time- and frequency-domain significantly decrease in T2DM patients with good glycemic control (HbA1c < 7.0%) and poor glycemic control (HbA1c > 7.0%), compared to healthy controls (Yu et al., 2020).

In this study, significant associations were only found between MSE complexity during sleep period and HbA1c, and significant group differences between subjects that had poor control of diabetes (HbA1c ≥ 9.0%, *n* = 10) and did not (HbA1c < 9.0%, *n* = 16) were observed in many non-linear metrics during unstable and stable sleep. Moreover, non-linear HRV metrics during sleep were significantly associated with FBG level, which is one of the important indicators for glycemic compliance (Association, 2013). The results indicates that non-linear HRV metrics during sleep may better manifest neuronal dysfunction caused by hyperglycemia, which induces oxidative stress and toxic glycosylation products (Pop-Busui, 2010), highlighting the importance of non-linear analysis during sleep.

Interestingly, higher HbA1c level was associated with higher entropy related metrics (see Table 8), the elevation of complexity may indicate worse metabolic function due to poor diabetic control (Costa et al., 2005). Additionally, in each sleep stage, the correlations between non-linear HRV metrics and FBG changed significantly before and after hospitalization, possibly due to the immediate impact of hospitalization on blood glucose control, reflecting the sensitivity of non-linear HRV assessment on glycemic control.

Due to the complex interaction among sleep, diabetic control and ANS function that sleep have a significant effect on both ANS and metabolic function, especially glycemic control of T2DM patients (Koren et al., 2011; Jordan et al., 2014; Kent et al., 2014; Martyn-Nemeth et al., 2018; Hur et al., 2020), partial correlation analysis was required to manifest the association between non-linear HRV metrics in sleep stages and glycemic control indicators clearly. With the nullification of sleep quality metrics, most of the correlations retained, which supported the significant correlations acquired by simple correlation analysis. Whereas the association significantly decreased or even vanished when TST was corrected, indicating the significant role of sleep quality in adjusting ANS and glycemic function of T2DM patients (Jordan et al., 2014; Martyn-Nemeth et al., 2018). Additionally, when stable sleep related metrics, especially SST, were nullified, the correlation between non-linear HRV metrics in sleep stages and glycemic control indicators significantly strengthened, demonstrating the functional significance of stable sleep stage. As well as stable sleep stage (slow wave sleep) is widely recognized as the most restorative of all sleep stages (Van Cauter et al., 2008) and when several important physiological activities occur specifically (Somers et al., 1993), researchers have found that stable sleep associates with brain glucose metabolism (Zoccoli et al., 2002) and suppression of stable sleep without any reduction in TST associates with poor diabetic control and increased risk of T2DM (Tasali et al., 2008).

In our original study, we also used RR interval time series from four time periods (late night, 3:00–4:00; dawn, 6:00–7:00; after lunch, 13:00–14:00; after dinner, 19:00–20:00) to perform HRV analysis and correlation analysis with metabolic indicators (see Supplementary Table S4–S7). Although distinct correlations were found between HRV metrics and metabolic functions during specific time periods, such as correlations between HRV and BUN, DBP or Hb at dawn, between HRV and HDL-C or LDL-C after lunch, and between metabolic indicators and LF/HF or LFP after dinner, the overall results were not satisfactory and systematic enough to be included in the main text. This may be due to the different physiological states of individuals at specific time periods and is influenced by meals and daytime activities.

To the best of our knowledge, few studies have applied time-, frequency-domain and non-linear analysis on HRV sequences in each sleep stage to inquire the association among ANS function, sleep cycle and metabolic function. We compared our results with those of using traditional methods in all-night sleep analysis and short-term linear HRV analysis. Representative results are shown in Table 11 in summary. In comparison with studies of correlations between all-night sleep and metabolic function, conducted among subjects without T2DM (Koren et al., 2011; Martyn-Nemeth et al., 2018; Feng et al., 2021), correlations found in this study were extensively stronger and broadened with a similar or notably smaller sample size. In comparison with studies of correlations between short-term linear HRV analysis and metabolic function, conducted among T2DM patients (Bhati et al., 2019), correlations found in this study were extensively stronger and broadened with a similar sample size, suggesting the potential of HRV analysis during sleep cycles in T2DM patients. However, when compared with a study with larger sample size (Balikai et al., 2022), correlations found in this study were more comprehensive but slightly weaker, which may be due to the limited sample size, exclusion of female subjects, differences in the physiological status of the subjects including duration and severity of diabetes since our subjects were all patients during hospitalization, the bias introduced by non-linear metrics including ApEn and MSE *via* the contamination of variance and suboptimal filtering procedure (Pincus and Huang, 1992; Nikulin and Brismar, 2004; Porta et al., 2007a; Valencia et al., 2009; Delgado-Bonal and Marshak, 2019) and the selection of long-term HRV sequences since metrics including LF, HF and SDNN would be affected by length of the sequences (Saul et al., 1988; Furlan et al., 1990; Task Force of the European Society of Cardiology the North American Society of Pacing Electrophysiology, 1996).

**TABLE 11:** Comparison of related studies on the interactions among sleep, HRV and metabolic function: subjects, methods, results and conclusion.

	Subjects	Method	Results	Conclusion
Martyn-Nemeth et al., (2018)	48 T1DM patients.	Group difference analysis on glycemic control according to the PSQI score (≤5 for good sleep quality and > 5 for poor sleep quality).	Poor sleep quality associated with greater nocturnal glycemic variability (*p* = 0.02). HbA1c did not vary between sleep quality groups.	Nocturnal glycemic variability associated with poor sleep quality.
Koren et al. (2011)	62 obese pubertal adolescents.	Analysis on the correlations between sleep architecture through overnight polysomnography, glucose and insulin homeostasis.	Significant associations between sleep duration and HbA1c (TST, *r* = −0.36, *p* < 0.01; REM duration, *r* = −0.35, *p* < 0.01) and fasting plasma glucose (N3 duration, *r* = −0.33, *p* < 0.01; REM duration, *r* = −0,31, *p* < 0.017).	There were significant relationships between sleep architecture and measures of glucose homeostasis and insulin secretion.
Feng et al. (2021)	2,308 suspected OSA patients.	Analysis on the correlations between sleep architecture through overnight polysomnography andclinical indicators (glucose, insulin, blood pressure, TG, HDL-C and LDL-C).	Significant weak correlations were found between SWS/TST ratio and BMI (*r* = −0.12, *p* < 0.01); between sleep efficiency and BMI (*r* = 0.11, *p* < 0.01); between MAI and BMI (*r* = 0.23, *p* < 0.01), glucose (*r* = 0.15, *p* < 0.01), insulin (*r* = 0.23, *p* < 0.01), DBP (*r* = 0.14, *p* < 0.01), TC (*r* = 0.13, *p* < 0.01), TG (*r* = 0.15, *p* < 0.01), LDL (*r* = 0.18, *p* < 0.01).	Only weak associations were observed between sleep and clinical indicators.
Bhati et al. (2019)	50 T2DM patients.	Analysis on the correlations between cardiac autonomic control evaluated by time-frequency domain HRV of 5-min ECG recorded after a rest period in the supine position and clinical indicators of inflammatory (IL-6, IL-18 and hsCRP) and endothelial function (NO, eNOS and ET-1).	Significant associations were found between IL-6 and TP (*r* = −0.34, *p* = 0.01), LF (*r* = −0.36, *p* < 0.01); between IL-18 and pNN50 (*r* = −0.53, *p* < 0.01), HF (*r* = −0.34, *p* < 0.05); between hsCRP and RMSSD (*r* = −0.40, *p* < 0.01), TP (*r* = −0.35, *p* = 0.01), LF (*r* = −0.31, *p* < 0.05), HF (*r* = −0.42, *p* < 0.01); between NO and RMSSD (*r* = 0.65, *p* < 0.001), TP (*r* = 0.30, *p* < 0.05), HF(*r* = 0.32, *p* < 0.05); between eNOS and RMSSD (*r* = 0.43, *p* < 0.01). Whereas, ET-1 is not associated with any HRV indices.	Clinical indicators of inflammation and endothelial function are associated with global HRV, suggesting pathophysiological link between subclinical inflammation, endothelial dysfunction and cardiac autonomic dysfunction in T2DM.
Balikai et al. (2022)	120 T2DM patients.	Analysis on the correlations between one-minute HRV time-frequency domain indices during forced deep breathing and fasting serum HDL levels.	Significant correlations were observed between HDL and Mean HR (*r* = 0.74, *p* < 0.001), LFnu (relative power in normal units, *r* = −0.62, *p* < 0.001) and ratio of LF/HF (*r* = 0.53, *p* < 0.001); between HDL and HFnu (relative power in normal units, *r* = 0.64, *p* < 0.001), SDNN (*r* = 0.75, *p* < 0.001), RMSSD (*r* = 0.63, *p* < 0.001) and pNN50 (*r* = 0.82, *p* < 0.001).	HDL-C level and all other HRV indices are dependent on each other in patients with T2DM. And most of the patients with low HDL-C level might be associated with autonomic imbalance.
Our study	64 T2DM patients	Analysis on the correlations between sleep quality, 24-h/awake/sleep/sleep staging HRV and clinical indicators of metabolic function.	Significant correlations were found between sleep quality and metabolic function (admission FBG, DBP, WBC, Hb, ALT, AST/ALT, BUN, UA, TG, HDL-C, LDL-C, UACR, |r| = 0.386 ± 0.062, *p* < 0.05); HRV derived ANS function showed strengthened correlations with metabolic function during sleep period (admission FBG, DBP, Hb, ALT, AST/ALT, GGT, BUN, UACR, |r| = 0.474 ± 0.100, *p* < 0.05); non-linear HRV metrics during sleep stages coupled more tightly with clinical indicators of metabolic function than linear analysis (HbA1c, DBP, N%, Hb, ALT, AST, AST/ALT, GGT, BUN, LDL-C, UMA, UACR, |r| = 0.462 ± 0.086, *p* < 0.05), and showed significant association with glycemic control.	HRV metrics during sleep period plays more distinct role than during awake period in investigating ANS dysfunction and metabolism in T2DM patients, and sleep rhythm based HRV analysis should perform better in ANS and metabolic function assessment, especially for glycemic control in non-linear analysis among T2DM patients.

Therefore, this study provides new insights into research on the interaction among sleep, ANS and metabolic function, for T2DM patients, especially for diabetic control. Researchers have found that the dysfunction of T2DM patients’ ANS manifests first in decreased parasympathetic activity (Pfeifer et al., 1982; Singh et al., 2000; Goit et al., 2012), even before clinical symptoms. The effective use of HRV analysis during sleep cycles to extract dynamic features of PNS and SNS in each sleep stage provides support for early detection of autonomic neuropathy. In addition, the extensively strong correlations between non-linear HRV metrics and glycemic control indicators including FBG and HbA1c demonstrates the validity of HRV analysis in each sleep stage, which can be another promising strategy for diabetic control and early diagnose of T2DM with less computational costs.

For further research, this study proposed to select HRV sequences based on physiological rhythms can locally reveal the dynamic features of HRV and magnify the potential biological origins and kinetic mechanisms of HRV under the influence of many external factors, such as respiration, circadian rhythm, sympathetic and vagal activity, as well as intrinsic factors, such as ion channel fluctuations and other molecular fluctuations, while reducing the computational cost and elevating local resolution of long term signals, providing new insights into long term physiological signal processing.

There are several potential limitations of this study. Mainly middle-aged male inpatients were included in the analysis, who do not represent the whole spectrum of T2DM patients, to eliminate the influence of climacterium on metabolic function. Although we did a further outlier rejection to reduce the influences of specific situations, there are still diversities between people with different physiological factors (age, gender, health status, course of disease, etc.) that were not included in this study, requiring further studies. Despite of the non-linear dynamics in HRV sequences appeared in our study, researchers have found that short-term heart period variability in healthy young adults at rest is mainly linear (Porta et al., 2007b). Since there weren’t thorough restrictions on subjects’ daily behaviour during hospitalization, further studies are required to investigate the production of non-linear components in long-term HRV sequences by applying forcing input to subjects. Lastly, due to the limited sample size, we did not build more accurate statistical models of HRV, ANS, sleep and metabolic function to further investigate their potential biological origins and kinetic mechanisms.

## Data Availability

The raw data supporting the conclusion of this article will be made available by the authors, without undue reservation.

## References

[B1] Akselrod S. Gordon D. Ubel F. A. Shannon D. C. Berger A. C. Cohen R. J. (1981). Power spectrum analysis of heart rate fluctuation: A quantitative probe of beat-to-beat cardiovascular control. Science 213(4504), 220–222. 10.1126/science.6166045 6166045

[B2] Association A. D. (2013). Diagnosis and classification of diabetes mellitus. Diabetes Care 37 (1), S81–S90. 10.2337/dc14-S081 24357215

[B3] Azami H. Escudero J. (2016). Matlab codes for "refined multiscale fuzzy entropy based on standard deviation for biomedical signal analysis. University of Edinburgh, School of Engineering, Institute for Digital Communications. 10.1007/s11517-017-1647-5 PMC5644759 28462498

[B4] Balbo M. Leproult R. Van Cauter E. (2010). Impact of sleep and its disturbances on hypothalamo-pituitary-adrenal Axis activity. Int. J. Endocrinol. 2010, 759234. 10.1155/2010/759234 20628523 PMC2902103

[B5] Balcıoğlu A. S. Müderrisoğlu H. (2015). Diabetes and cardiac autonomic neuropathy: Clinical manifestations, cardiovascular consequences, diagnosis and treatment. World J. Diabetes 6 (1), 80–91. 10.4239/wjd.v6.i1.80 25685280 PMC4317320

[B6] Balikai F. A. Javali S. B. Shindhe V. M. Deshpande N. Benni J. M. Shetty D. P. (2022). Correlation of serum HDL level with HRV indices using multiple linear regression analysis in patients with type 2 diabetes mellitus. Diabetes Res. Clin. Pract. 190, 109988. 10.1016/j.diabres.2022.109988 35835257

[B7] Barone M. T. U. Menna-Barreto L. (2011). Diabetes and sleep: A complex cause-and-effect relationship. Diabetes Res. Clin. Pract. 91 (2), 129–137. 10.1016/j.diabres.2010.07.011 20810183

[B8] Bhati P. Alam R. Moiz J. A. Hussain M. E. (2019). Subclinical inflammation and endothelial dysfunction are linked to cardiac autonomic neuropathy in type 2 diabetes. J. Diabetes & Metabolic Disord. 18 (2), 419–428. 10.1007/s40200-019-00435-w PMC6915244 31890667

[B9] Bigger J. T. Jr. Albrecht P. Steinman R. C. Rolnitzky L. M. Fleiss J. L. Cohen R. J. (1989). Comparison of time- and frequency domain-based measures of cardiac parasympathetic activity in Holter recordings after myocardial infarction. Am. J. Cardiol. 64 (8), 536–538. 10.1016/0002-9149(89)90436-0 2773799

[B10] Brandenberger G. Ehrhart J. Piquard F. Simon C. (2001). Inverse coupling between ultradian oscillations in delta wave activity and heart rate variability during sleep. Clin. Neurophysiol. 112 (6), 992–996. 10.1016/S1388-2457(01)00507-7 11377256

[B11] Buysse D. J. Reynolds C. F. Monk T. H. Berman S. R. Kupfer D. J. (1989). The Pittsburgh sleep quality index: A new instrument for psychiatric practice and research. Psychiatry Res. 28 (2), 193–213. 10.1016/0165-1781(89)90047-4 2748771

[B12] Cerutti S. Esposti F. Ferrario M. Sassi R. Signorini M. G. (2007). Long-term invariant parameters obtained from 24-h holter recordings: A comparison between different analysis techniques. Chaos An Interdiscip. J. Nonlinear Sci. 17 (1), 015108. 10.1063/1.2437155 17411265

[B13] Chen W. Zhuang J. Yu W. Wang Z. (2009). Measuring complexity using FuzzyEn, ApEn, and SampEn. Med. Eng. Phys. 31 (1), 61–68. 10.1016/j.medengphy.2008.04.005 18538625

[B15] Cooke W. H. Hoag J. B. Crossman A. A. Kuusela T. A. Tahvanainen K. U. O. Eckberg D. L. (1999). Human responses to upright tilt: A window on central autonomic integration. J. Physiology 517 (2), 617–628. 10.1111/j.1469-7793.1999.0617t.x PMC2269357 10332107

[B16] Costa M. Goldberger A. L. Peng C. K. (2005). Multiscale entropy analysis of biological signals. Phys. Rev. E 71 (2), 021906. 10.1103/PhysRevE.71.021906 15783351

[B17] Costa M. Goldberger A. L. Peng C. K. (2002). Multiscale entropy analysis of complex physiologic time series. Phys. Rev. Lett. 89 (6), 068102. 10.1103/PhysRevLett.89.068102 12190613

[B18] Cui X. Tian L. Li Z. Ren Z. Zha K. Wei X. (2020). On the variability of heart rate variability—evidence from prospective study of healthy young college students. Entropy 22 (11), 1302. 10.3390/e22111302 33263356 PMC7711844

[B19] Cuschieri S. (2019). The STROBE guidelines. Saudi J. Anaesth. 13 (1), S31–s34. 10.4103/sja.SJA_543_18 30930717 PMC6398292

[B20] Cysarz D. Bettermann H. Leeuwen P. v. (2000). Entropies of short binary sequences in heart period dynamics. Am. J. Physiology-Heart Circulatory Physiology 278 (6), H2163–H2172. 10.1152/ajpheart.2000.278.6.H2163 10843917

[B21] de Moura-Tonello S. C. G. Porta A. Marchi A. de Almeida Fagundes A. Francisco C. d. O. Rehder-Santos P. (2016). Cardiovascular variability analysis and baroreflex estimation in patients with type 2 diabetes in absence of any manifest neuropathy. PLOS ONE 11 (3), e0148903. 10.1371/journal.pone.0148903 26987126 PMC4795601

[B22] de Zambotti M. Trinder J. Silvani A. Colrain I. M. Baker F. C. (2018). Dynamic coupling between the central and autonomic nervous systems during sleep: A review. Neurosci. Biobehav. Rev. 90, 84–103. 10.1016/j.neubiorev.2018.03.027 29608990 PMC5993613

[B23] Delgado-Bonal A. Marshak A. (2019). Approximate entropy and sample entropy: A comprehensive tutorial. Entropy 21 (6), 541. 10.3390/e21060541 33267255 PMC7515030

[B24] Dohare A. K. Kumar V. Kumar R. (2014). An efficient new method for the detection of QRS in electrocardiogram. Comput. Electr. Eng. 40 (5), 1717–1730. 10.1016/j.compeleceng.2013.11.004

[B25] Faust O. Acharya U. R. Molinari F. Chattopadhyay S. Tamura T. (2012). Linear and non-linear analysis of cardiac health in diabetic subjects. Biomed. Signal Process. Control 7 (3), 295–302. 10.1016/j.bspc.2011.06.002

[B26] Fei L. Copie X. Malik M. Camm A. J. (1996). Short- and long-term assessment of heart rate variability for risk stratification after acute myocardial infarction. Am. J. Cardiol. 77 (9), 681–684. 10.1016/S0002-9149(97)89199-0 8651116

[B27] Feng N. Yang J. Xu H. Zhang C. Wang F. Wu X. (2021). The associations between sleep architecture and metabolic parameters in patients with obstructive sleep apnea: A hospital-based cohort study. Front. Neurology 12, 606031. 10.3389/fneur.2021.606031 PMC7919522 33658975

[B28] Francis D. P. Willson K. Georgiadou P. Wensel R. Davies L. C. Coats A. (2002). Physiological basis of fractal complexity properties of heart rate variability in man. J. Physiology 542 (2), 619–629. 10.1113/jphysiol.2001.013389 PMC2290419 12122157

[B29] Frattola A. Parati G. Gamba P. Paleari F. Mauri G. Di Rienzo M. (1997). Time and frequency domain estimates of spontaneous baroreflex sensitivity provide early detection of autonomic dysfunction in diabetes mellitus. Diabetologia 40 (12), 1470–1475. 10.1007/s001250050851 9447956

[B30] Furlan R. Guzzetti S. Crivellaro W. Dassi S. Tinelli M. Baselli G. (1990). Continuous 24-hour assessment of the neural regulation of systemic arterial pressure and RR variabilities in ambulant subjects. Circulation 81(2), 537–547. 10.1161/01.CIR.81.2.537 2297860

[B31] Garber A. J. Handelsman Y. Grunberger G. Einhorn D. Abrahamson M. J. Barzilay J. I. (2020). Consensus statement by the American association of clinical endocrinologists and American college of endocrinology on the comprehensive type 2 diabetes management algorithm – 2020 executive summary. Endocr. Pract. 26 (1), 107–139. 10.4158/CS-2019-0472 32022600

[B32] Gerritsen J. Dekker J. M. TenVoorde B. J. Kostense P. J. Heine R. J. Bouter L. M. (2001). Impaired autonomic function is associated with increased mortality, especially in subjects with diabetes, hypertension, or a history of cardiovascular disease: The hoorn study. Diabetes Care 24 (10), 1793–1798. 10.2337/diacare.24.10.1793 11574444

[B33] Glos M. Fietze I. Blau A. Baumann G. Penzel T. (2014). Cardiac autonomic modulation and sleepiness: Physiological consequences of sleep deprivation due to 40h of prolonged wakefulness. Physiology Behav. 125, 45–53. 10.1016/j.physbeh.2013.11.011 24291386

[B34] Goit R. K. Khadka R. Sharma S. K. Limbu N. Paudel B. H. (2012). Cardiovascular autonomic function and vibration perception threshold in type 2 diabetes mellitus. J. Diabetes its Complicat. 26 (4), 339–342. 10.1016/j.jdiacomp.2012.03.026 22534513

[B35] González J. J. Cordero J. J. Feria M. Pereda E. (2000). Detection and sources of nonlinearity in the variability of cardiac R-R intervals and blood pressure in rats. Am. J. Physiology-Heart Circulatory Physiology 279 (6), H3040–H3046. 10.1152/ajpheart.2000.279.6.H3040 11087262

[B36] Hamilton P. S. Tompkins W. J. (1986). Quantitative investigation of QRS detection rules using the MIT/BIH arrhythmia database. Ieee Trans. Biomed. Eng. 33 (12), 1157–1165. 10.1109/tbme.1986.325695 3817849

[B37] Hur M. H. Lee M. K. Seong K. Hong J. H. (2020). Deterioration of sleep quality according to glycemic status. Diabetes & Metabolism J. 44 (5), 679–686. 10.4093/dmj.2019.0125 PMC7643591 32431108

[B38] Inzucchi S. E. Bergenstal R. M. Buse J. B. Diamant M. Ferrannini E. Nauck M. (2015). Management of hyperglycemia in type 2 diabetes, 2015: A patient-centered approach: Update to a position statement of the American diabetes association and the European association for the study of diabetes. Diabetes Care 38 (1), 140–149. 10.2337/dc14-2441 25538310

[B39] Jo J. A. Blasi A. Valladares E. Juarez R. Baydur A. Khoo M. C. K. (2005). Determinants of heart rate variability in obstructive sleep apnea syndrome during wakefulness and sleep. Am. J. Physiology-Heart Circulatory Physiology 288 (3), H1103–H1112. 10.1152/ajpheart.01065.2003 15471971

[B40] Johnson A. E. W. Behar J. Andreotti F. Clifford G. D. Oster J. (2015). Multimodal heart beat detection using signal quality indices. Physiol. Meas. 36 (8), 1665–1677. 10.1088/0967-3334/36/8/1665 26218060

[B41] Jordan A. S. McSharry D. G. Malhotra A. (2014). Adult obstructive sleep apnoea. Lancet 383 (9918), 736–747. 10.1016/s0140-6736(13)60734-5 23910433 PMC3909558

[B42] Kent B. D. Grote L. Ryan S. Pépin J. L. Bonsignore M. R. Tkacova R. (2014). Diabetes mellitus prevalence and control in sleep-disordered breathing: The European sleep apnea cohort (ESADA) study. Chest 146 (4), 982–990. 10.1378/chest.13-2403 24831859

[B43] Kleiger R. E. Stein P. K. Bigger J. T. Jr. (2005). Heart rate variability: Measurement and clinical utility. Ann. Noninvasive Electrocardiol. 10 (1), 88–101. 10.1111/j.1542-474X.2005.10101.x 15649244 PMC6932537

[B44] Koren D. Levitt Katz L. E. Brar P. C. Gallagher P. R. Berkowitz R. I. Brooks L. J. (2011). Sleep architecture and glucose and insulin homeostasis in obese adolescents. Diabetes Care 34 (11), 2442–2447. 10.2337/dc11-1093 21933909 PMC3198280

[B45] Laborde S. Mosley E. Thayer J. F. (2017). Heart rate variability and cardiac vagal tone in psychophysiological research – recommendations for experiment planning, data analysis, and data reporting. Front. Psychol. 8, 213. 10.3389/fpsyg.2017.00213 28265249 PMC5316555

[B46] Leproult R. Copinschi G. Buxton O. Van Cauter E. (1997). Sleep loss results in an elevation of cortisol levels the next evening. Sleep 20 (10), 865–870. 10.1093/sleep/20.10.865 9415946

[B47] Li F. Yang H. (2013). A real-time QRS complex detection algorithm based on composite filter and curve feature point extraction. Comput. Appl. Softw. 30 (11).

[B48] Liu S.-H. Lo L.-W. Tsai T.-Y. Cheng W.-H. Lin Y.-J. Chang S.-L. (2020). Circadian rhythm dynamics on multiscale entropy identifies autonomic dysfunction associated with risk of ventricular arrhythmias and near syncope in chronic kidney disease. J. Cardiol. 76 (6), 542–548. 10.1016/j.jjcc.2020.05.017 32631644

[B49] Lombardi F. Stein P. K. (2011). Origin of heart rate variability and turbulence: An appraisal of autonomic modulation of cardiovascular function. Front. Physiology 2, 95. 10.3389/fphys.2011.00095 PMC3233900 22163222

[B50] López-Cano C. Gutiérrez-Carrasquilla L. Sánchez E. González J. Yeramian A. Martí R. (2019). Sympathetic hyperactivity and sleep disorders in individuals with type 2 diabetes. Front. Endocrinol. 10, 752. 10.3389/fendo.2019.00752 PMC6839128 31736881

[B51] Mador M. J. Tobin M. J. (1991). Effect of alterations in mental activity on the breathing pattern in healthy subjects. Am. Rev. Respir. Dis. 144 (3), 481–487. 10.1164/ajrccm/144.3_Pt_1.481 1892283

[B52] Magagnin V. Bassani T. Bari V. Turiel M. Maestri R. Pinna G. D. (2011). Non-stationarities significantly distort short-term spectral, symbolic and entropy heart rate variability indices. Physiol. Meas. 32 (11), 1775–1786. 10.1088/0967-3334/32/11/S05 22027399

[B53] Malliani A. Pagani M. Lombardi F. Cerutti S. (1991). Cardiovascular neural regulation explored in the frequency domain. Circulation 84(2), 482–492. 10.1161/01.CIR.84.2.482 1860193

[B54] Marcheva B. Ramsey K. M. Buhr E. D. Kobayashi Y. Su H. Ko C. H. (2010). Disruption of the clock components CLOCK and BMAL1 leads to hypoinsulinaemia and diabetes. Nature 466 (7306), 627–631. 10.1038/nature09253 20562852 PMC2920067

[B55] Markwald R. R. Melanson E. L. Smith M. R. Higgins J. Perreault L. Eckel R. H. (2013). Impact of insufficient sleep on total daily energy expenditure, food intake, and weight gain. Proc. Natl. Acad. Sci. 110(14), 5695–5700. 10.1073/pnas.1216951110 23479616 PMC3619301

[B56] Markwald R. R. Wright K. P. (2012). “Circadian misalignment and sleep disruption in shift work: Implications for fatigue and risk of weight gain and obesity,” in Sleep loss and obesity: Intersecting epidemics. Editors Shiromani P. Horvath T. Redline S. Van Cauter E. (New York, NY: Springer New York), 101–118.

[B57] Martelli D. Silvani A. McAllen R. M. May C. N. Ramchandra R. (2014). The low frequency power of heart rate variability is neither a measure of cardiac sympathetic tone nor of baroreflex sensitivity. Am. J. Physiology-Heart Circulatory Physiology 307 (7), H1005–H1012. 10.1152/ajpheart.00361.2014 25063795

[B58] Martyn-Nemeth P. Phillips S. A. Mihailescu D. Farabi S. S. Park C. Lipton R. (2018). Poor sleep quality is associated with nocturnal glycaemic variability and fear of hypoglycaemia in adults with type 1 diabetes. J. Adv. Nurs. 74 (10), 2373–2380. 10.1111/jan.13765 29917259 PMC7778470

[B59] Maser R. E. Mitchell B. D. Vinik A. I. Freeman R. (2003). The association between cardiovascular autonomic neuropathy and mortality in individuals with diabetes: A meta-analysis. Diabetes Care 26 (6), 1895–1901. 10.2337/diacare.26.6.1895 12766130

[B60] Montano N. Porta A. Cogliati C. Costantino G. Tobaldini E. Casali K. R. (2009). Heart rate variability explored in the frequency domain: A tool to investigate the link between heart and behavior. Neurosci. Biobehav. Rev. 33 (2), 71–80. 10.1016/j.neubiorev.2008.07.006 18706440

[B61] Mosenzon O. Pollack R. Raz I. (2016). Treatment of type 2 diabetes: From "guidelines" to "position statements" and back: Recommendations of the Israel national diabetes council. Diabetes Care 39 (2), S146–S153. 10.2337/dcS15-3003 27440827

[B62] National Center for Cardiovascular Diseases, Chinese medical doctor association, hypertension committee of Chinese medical doctor association, Chinese society of Cardiology, Chinese medical association,, and hypertension committee of cross-straits medical exchanges association (2022). clinical practice guidelines for the management of hypertension in China. Chin. J. Cardiol. 50, 1050–1095. 10.3760/cma.j.cn112148-20220809-00613

[B63] Nguyen J. Wright K. P. Jr. (2010). Influence of weeks of circadian misalignment on leptin levels. Nat. Sci. Sleep. 2, 9–18. 10.2147/nss.s7624 23616693 PMC3603689

[B64] Nikulin V. V. Brismar T. (2004). Comment on "Multiscale entropy analysis of complex physiologic time series". Phys. Rev. Lett. 92 (8), 089803. author reply 089804. 10.1103/PhysRevLett.92.089803 14995828

[B65] Nolan J. Batin P. D. Andrews R. Lindsay S. J. Brooksby P. Mullen M. (1998). Prospective study of heart rate variability and mortality in chronic heart failure: Results of the United Kingdom heart failure evaluation and assessment of risk trial (UK-heart). Circulation 98(15), 1510–1516. 10.1161/01.CIR.98.15.1510 9769304

[B66] Nunan D. Sandercock G. R. H. Brodie D. A. (2010). A quantitative systematic review of normal values for short-term heart rate variability in healthy adults. Pacing Clin. Electrophysiol. 33 (11), 1407–1417. 10.1111/j.1540-8159.2010.02841.x 20663071

[B67] Pagani M. Lombardi F. Guzzetti S. Rimoldi O. Furlan R. Pizzinelli P. (1986). Power spectral analysis of heart rate and arterial pressure variabilities as a marker of sympatho-vagal interaction in man and conscious dog. Circulation Res. 59(2), 178–193. 10.1161/01.RES.59.2.178 2874900

[B68] Pan J. Tompkins W. J. (1985). A real-time QRS detection algorithm. Ieee Trans. Biomed. Eng. 32 (3), 230–236. 10.1109/tbme.1985.325532 3997178

[B69] Peng C. K. Buldyrev S. V. Havlin S. Simons M. Stanley H. E. Goldberger A. L. (1994). Mosaic organization of DNA nucleotides. Phys. Rev. E 49 (2), 1685–1689. 10.1103/PhysRevE.49.1685 9961383

[B70] Peng C. K. Havlin S. Stanley H. E. Goldberger A. L. (1995). Quantification of scaling exponents and crossover phenomena in nonstationary heartbeat time series. Chaos An Interdiscip. J. Nonlinear Sci. 5 (1), 82–87. 10.1063/1.166141 11538314

[B71] Perkiömäki J. (2011). Heart rate variability and non-linear dynamics in risk stratification. Front. Physiology 2, 81. 10.3389/fphys.2011.00081 PMC3210967 22084633

[B72] Pfeifer M. A. Cook D. Brodsky J. Tice D. Reenan A. Swedine S. (1982). Quantitative evaluation of cardiac parasympathetic activity in normal and diabetic man. Diabetes 31 (4), 339–345. 10.2337/diab.31.4.339 7152130

[B73] Pincus S. M. (1991). Approximate entropy as a measure of system complexity. Proc. Natl. Acad. Sci. 88(6), 2297–2301. 10.1073/pnas.88.6.2297 11607165 PMC51218

[B74] Pincus S. M. Cummins T. R. Haddad G. G. (1993). Heart rate control in normal and aborted-SIDS infants. Am. J. Physiology-Regulatory, Integr. Comp. Physiology 264 (3), R638–R646. 10.1152/ajpregu.1993.264.3.R638 8457020

[B75] Pincus S. M. Huang W.-M. (1992). Approximate entropy: Statistical properties and applications. Commun. Statistics - Theory Methods 21 (11), 3061–3077. 10.1080/03610929208830963

[B76] Pop-Busui R. (2010). Cardiac autonomic neuropathy in diabetes: A clinical perspective. Diabetes Care 33 (2), 434–441. 10.2337/dc09-1294 20103559 PMC2809298

[B77] Porta A. Baselli G. Guzzetti S. Pagani M. Malliani A. Cerutti S. (2000a). Prediction of short cardiovascular variability signals based on conditional distribution. IEEE Trans. Biomed. Eng. 47 (12), 1555–1564. 10.1109/10.887936 11125590

[B78] Porta A. Gnecchi-Ruscone T. Tobaldini E. Guzzetti S. Furlan R. Montano N. (2007a). Progressive decrease of heart period variability entropy-based complexity during graded head-up tilt. J. Appl. Physiology 103 (4), 1143–1149. 10.1152/japplphysiol.00293.2007 17569773

[B79] Porta A. Guzzetti S. Furlan R. Gnecchi-Ruscone T. Montano N. Malliani A. (2007b). Complexity and nonlinearity in short-term heart period variability: Comparison of methods based on local nonlinear prediction. IEEE Trans. Biomed. Eng. 54 (1), 94–106. 10.1109/TBME.2006.883789 17260860

[B80] Porta A. Guzzetti S. Montano N. Furlan R. Pagani M. Malliani A. (2001). Entropy, entropy rate, and pattern classification as tools to typify complexity in short heart period variability series. IEEE Trans. Biomed. Eng. 48 (11), 1282–1291. 10.1109/10.959324 11686627

[B81] Porta A. Guzzetti S. Montano N. Pagani M. Somers V. Malliani A. (2000b). Information domain analysis of cardiovascular variability signals: Evaluation of regularity, synchronisation and co-ordination. Med. Biol. Eng. Comput. 38 (2), 180–188. 10.1007/bf02344774 10829411

[B82] Porta A. Maria B. D. Bari V. Marchi A. Faes L. (2017). Are nonlinear model-free conditional entropy approaches for the assessment of cardiac control complexity superior to the linear model-based one? IEEE Trans. Biomed. Eng. 64 (6), 1287–1296. 10.1109/TBME.2016.2600160 27541327

[B83] Punjabi N. M. Polotsky V. Y. (2005). Disorders of glucose metabolism in sleep apnea. J. Appl. Physiology 99 (5), 1998–2007. 10.1152/japplphysiol.00695.2005 16227461

[B84] Reyes del Paso G. A. Langewitz W. Mulder L. J. M. van Roon A. Duschek S. (2013). The utility of low frequency heart rate variability as an index of sympathetic cardiac tone: A review with emphasis on a reanalysis of previous studies. Psychophysiology 50 (5), 477–487. 10.1111/psyp.12027 23445494

[B85] Richman J. S. Moorman J. R. (2000). Physiological time-series analysis using approximate entropy and sample entropy. Am. J. Physiology-Heart Circulatory Physiology 278 (6), H2039–H2049. 10.1152/ajpheart.2000.278.6.H2039 10843903

[B86] Ruiz J. Monbaron D. Parati G. Perret S. Haesler E. Danzeisen C. (2005). Diabetic neuropathy is a more important determinant of baroreflex sensitivity than carotid elasticity in type 2 diabetes. Hypertension 46(1), 162–167. 10.1161/01.HYP.0000169053.14440.7d 15928031

[B87] Sako T. Burioka N. Suyama H. Nomura T. Takeshima T. Shimizu E. (2001). Nonlinear behavior of human respiratory movement during different sleep stages. Chronobiology Int. 18 (1), 71–83. 10.1081/CBI-100001172 11247115

[B88] Sassi R. Cerutti S. Lombardi F. Malik M. Huikuri H. V. Peng C.-K. (2015). Advances in heart rate variability signal analysis: Joint position statement by the e-cardiology ESC working group and the European heart rhythm association co-endorsed by the Asia pacific heart rhythm society. EP Eur. 17 (9), 1341–1353. 10.1093/europace/euv015 26177817

[B89] Saul J. P. Albrecht P. Berger R. D. Cohen R. J. (1988). Analysis of long term heart rate variability: Methods, 1/f scaling and implications. Comput. Cardiol. 14, 419–422. 11542156

[B90] Scheer F. A. J. L. Hilton M. F. Mantzoros C. S. Shea S. A. (2009). Adverse metabolic and cardiovascular consequences of circadian misalignment. Proc. Natl. Acad. Sci. 106(11), 4453–4458. 10.1073/pnas.0808180106 19255424 PMC2657421

[B91] Scholz U. J. Bianchi A. M. Cerutti S. Kubicki S. (1997). Vegetative background of sleep: Spectral analysis of the heart rate variability. Physiology Behav. 62 (5), 1037–1043. 10.1016/S0031-9384(97)00234-5 9333197

[B92] Schramm P. Neville A. G. Madison S. Thomas R. J. Baker D. N. (2009). Cardio-pulmonary coupling (CPC) measures correlate to standard sleep variables in a random clinical sample of patients suspected with sleep disordered breathing (SDB). Sleep 32, A189.

[B93] Shaffer F. McCraty R. Zerr C. L. (2014). A healthy heart is not a metronome: An integrative review of the heart's anatomy and heart rate variability. Front. Psychol. 5, 1040. 10.3389/fpsyg.2014.01040 25324790 PMC4179748

[B94] Silvani A. Dampney R. A. L. (2013). Central control of cardiovascular function during sleep. Am. J. Physiology-Heart Circulatory Physiology 305 (12), H1683–H1692. 10.1152/ajpheart.00554.2013 24097430

[B95] Singh J. P. Larson M. G. O'Donnell C. J. Wilson P. F. Tsuji H. Lloyd-Jones D. M. (2000). Association of hyperglycemia with reduced heart rate variability (The Framingham Heart Study). Am. J. Cardiol. 86 (3), 309–312. 10.1016/s0002-9149(00)00920-6 10922439

[B96] Solnik S. DeVita P. Rider P. Long B. Hortobágyi T. (2008). Teager-Kaiser Operator improves the accuracy of EMG onset detection independent of signal-to-noise ratio. Acta Bioeng. Biomech. 10 (2), 65–68. PMC2596643 19032000

[B97] Somers V. K. Dyken M. E. Mark A. L. Abboud F. M. (1993). Sympathetic-nerve activity during sleep in normal subjects. N. Engl. J. Med. 328 (5), 303–307. 10.1056/nejm199302043280502 8419815

[B98] Spiegel K. Knutson K. Leproult R. Tasali E. Cauter E. V. (2005). Sleep loss: A novel risk factor for insulin resistance and type 2 diabetes. J. Appl. Physiology 99 (5), 2008–2019. 10.1152/japplphysiol.00660.2005 16227462

[B99] Spiegel K. Leproult R. Van Cauter E. (1999). Impact of sleep debt on metabolic and endocrine function. Lancet 354 (9188), 1435–1439. 10.1016/S0140-6736(99)01376-8 10543671

[B100] Spiegel K. Tasali E. Leproult R. Scherberg N. Van Cauter E. (2011). Twenty-four-Hour profiles of acylated and total ghrelin: Relationship with glucose levels and impact of time of day and sleep. J. Clin. Endocrinol. Metabolism 96 (2), 486–493. 10.1210/jc.2010-1978 PMC3206394 21106712

[B101] Spira A. P. Beaudreau S. A. Stone K. L. Kezirian E. J. Lui L.-Y. Redline S. (2011). Reliability and validity of the Pittsburgh sleep quality index and the epworth sleepiness scale in older men. Journals Gerontology Ser. A 67A (4), 433–439. 10.1093/gerona/glr172 PMC3309871 21934125

[B102] Stein P. K. Pu Y. (2012). Heart rate variability, sleep and sleep disorders. Sleep. Med. Rev. 16 (1), 47–66. 10.1016/j.smrv.2011.02.005 21658979

[B103] Tank J. Jordan J. Neuke A. Mölle A. Weck M. (2001). Spontaneous baroreflex sensitivity and heart rate variability are not superior to classic autonomic testing in older patients with type 2 diabetes. Am. J. Med. Sci. 322 (1), 24–30. 10.1097/00000441-200107000-00005 11465243

[B104] Tasali E. Leproult R. Ehrmann D. A. Van Cauter E. (2008). Slow-wave sleep and the risk of type 2 diabetes in humans. Proc. Natl. Acad. Sci. 105(3), 1044–1049. 10.1073/pnas.0706446105 18172212 PMC2242689

[B105] Task Force of the European Society of Cardiology the North American Society of Pacing Electrophysiology (1996). Heart rate variability. Circulation 93(5), 1043–1065. 10.1161/01.CIR.93.5.1043 8598068

[B106] Thomas R. J. Mietus J. E. Peng C.-K. Goldberger A. L. (2005). An electrocardiogram-based technique to assess cardiopulmonary coupling during sleep. Sleep 28 (9), 1151–1161. 10.1093/sleep/28.9.1151 16268385

[B107] Thomas R. J. Mietus J. E. Peng C.-K. Goldberger A. L. Crofford L. J. Chervin R. D. (2010). Impaired sleep quality in fibromyalgia: Detection and quantification with ECG-based cardiopulmonary coupling spectrograms. Sleep. Med. 11 (5), 497–498. 10.1016/j.sleep.2009.09.003 20015685 PMC2854867

[B108] Tobaldini E. Costantino G. Solbiati M. Cogliati C. Kara T. Nobili L. (2017). Sleep, sleep deprivation, autonomic nervous system and cardiovascular diseases. Neurosci. Biobehav. Rev. 74, 321–329. 10.1016/j.neubiorev.2016.07.004 27397854

[B109] Tobaldini E. Pecis M. Montano N. (2014). Effects of acute and chronic sleep deprivation on cardiovascular regulation. Arch. Ital. De. Biol. 152 (2-3), 103–110. 10.12871/000298292014235 25828682

[B110] Trinder J. Kleiman J. Carrington M. Smith S. Breen S. Tan N. (2001). Autonomic activity during human sleep as a function of time and sleep stage. J. Sleep Res. 10 (4), 253–264. 10.1046/j.1365-2869.2001.00263.x 11903855

[B112] Turek F. W. Joshu C. Kohsaka A. Lin E. Ivanova G. McDearmon E. (2005). Obesity and metabolic syndrome in circadian *clock* mutant mice. Science 308(5724), 1043–1045. 10.1126/science.1108750 15845877 PMC3764501

[B113] Valencia J. F. Porta A. Vallverdu M. Claria F. Baranowski R. Orlowska-Baranowska E. (2009). Refined multiscale entropy: Application to 24-h holter recordings of heart period variability in healthy and aortic stenosis subjects. IEEE Trans. Biomed. Eng. 56 (9), 2202–2213. 10.1109/TBME.2009.2021986 19457745

[B114] Van Cauter E. Spiegel K. Tasali E. Leproult R. (2008). Metabolic consequences of sleep and sleep loss. Sleep. Med. 9, S23–S28. 10.1016/S1389-9457(08)70013-3 18929315 PMC4444051

[B115] VanCauter E. Polonsky K. S. Scheen A. J. (1997). Roles of circadian rhythmicity and sleep in human glucose regulation. Endocr. Rev. 18 (5), 716–738. 10.1210/er.18.5.716 9331550

[B116] Vinik A. I. Maser R. E. Mitchell B. D. Freeman R. (2003). Diabetic autonomic neuropathy. Diabetes Care 26 (5), 1553–1579. 10.2337/diacare.26.5.1553 12716821

[B117] Vinik A. I. Ziegler D. (2007). Diabetic cardiovascular autonomic neuropathy. Circulation 115(3), 387–397. 10.1161/CIRCULATIONAHA.106.634949 17242296

[B118] Voss A. Kurths J. Kleiner H. J. Witt A. Wessel N. Saparin P. (1996). The application of methods of non-linear dynamics for the improved and predictive recognition of patients threatened by sudden cardiac death. Cardiovasc Res. 31 (3), 419–433. 10.1016/0008-6363(96)00008-9 8681329

[B119] Wessel N. Ziehmann C. Kurths J. Meyerfeldt U. Schirdewan A. Voss A. (2000). Short-term forecasting of life-threatening cardiac arrhythmias based on symbolic dynamics and finite-time growth rates. Phys. Rev. E 61 (1), 733–739. 10.1103/PhysRevE.61.733 11046317

[B120] Yamada T. Katagiri H. (2007). Avenues of communication between the brain and tissues/organs involved in energy homeostasis. Endocr. J. 54 (4), 497–505. 10.1507/endocrj.KR-106 17510499

[B121] Ye S. Ruan P. Yong J. Shen H. Liao Z. Dong X. (2016). The impact of the HbA1c level of type 2 diabetics on the structure of haemoglobin. Sci. Rep. 6 (1), 33352. 10.1038/srep33352 27624402 PMC5022022

[B122] Yeh G. Y. Mietus J. E. Peng C.-K. Phillips R. S. Davis R. B. Wayne P. M. (2008). Enhancement of sleep stability with Tai Chi exercise in chronic heart failure: Preliminary findings using an ECG-based spectrogram method. Sleep. Med. 9 (5), 527–536. 10.1016/j.sleep.2007.06.003 17689142 PMC3281294

[B123] Yu Y. Hu L. Xu Y. Wu S. Chen Y. Zou W. (2020). Impact of blood glucose control on sympathetic and vagus nerve functional status in patients with type 2 diabetes mellitus. Acta Diabetol. 57 (2), 141–150. 10.1007/s00592-019-01393-8 31367992 PMC6997255

[B124] Zhang L. Wu H. Zhang X. Wei X. Hou F. Ma Y. (2020). Sleep heart rate variability assists the automatic prediction of long-term cardiovascular outcomes. Sleep. Med. 67, 217–224. 10.1016/j.sleep.2019.11.1259 31972509 PMC7281861

[B125] Ziegler D. Zentai C. P. Perz S. Rathmann W. Haastert B. Döring A. (2008). Prediction of mortality using measures of cardiac autonomic dysfunction in the diabetic and nondiabetic population: The MONICA/KORA augsburg cohort study. Diabetes Care 31 (3), 556–561. 10.2337/dc07-1615 18086873

[B126] Zoccoli G. Walker A. M. Lenzi P. Franzini C. (2002). The cerebral circulation during sleep: Regulation mechanisms and functional implications. Sleep. Med. Rev. 6 (6), 443–455. 10.1053/smrv.2001.0194 12505477

