# Supplementary material for: Heart rate variability in different sleep stages is associated with metabolic function and glycemic control in type 2 diabetes mellitus
Source: Front Physiol. 2023 Apr 14;14:1157270. doi: 10.3389/fphys.2023.1157270 (PMC10140569; doi:10.3389/fphys.2023.1157270)
Supplement: Supplementary file 1 [file DataSheet1.docx]

Supplementary Material

Heart rate variability in different sleep stages is associated with metabolic function and glycemic control in type 2 diabetes mellitus

Wenquan Cheng^†^, Hongsen Chen^†^, Leirong Tian, Zhimin Ma^*^, Xingran Cui^*^

*** Correspondence:** Xingran Cui: [cuixr@seu.edu.cn](mailto:cuixr@seu.edu.cn)

Zhimin Ma: [mazhimin01@sina.com](mailto:mazhimin01@sina.com)

†These authors contributed equally to this work and share first authorship

# Introductions of Non-linear HRV Analysis

It is more and more strongly suggested that the heart is not a periodic oscillator under normal physiologic conditions (Goldberger and West, 1987), and linear HRV metrics may fail detect subtle but important changes in heart rate time series. In our study, we utilized 2 kinds of non-linear HRV metrics: fractal measure, i.e., detrended fluctuation analysis (DFA, α1 and α2); entropy measures, including approximate entropy (ApEn), sample entropy (SampEn), fuzzy entropy (FuzzyEn), multiscale entropy (MSE) and multiscale fuzzy entropy (MFE). These metrics are widely acknowledged and used (Sassi et al., 2015).

## Detrended Fluctuation Analysis (DFA)

- 1. The entire RR series of length $N$ is divided into $N/L$ non-overlapping segments. In each segment, the number of RR intervals is $L$. A local trend is defined, which is the result of a linear least squares fit to the segment.
  2. Define the detrended signal $y_{L}(n)$ as the difference between the original signal $y(n)$ and the local trend.
  3. Calculate the variance of the detrended signal $y_{L}(n)$ for each segment and calculate the average of these variances for all segments of size $L$, denoted as $F(L)$.

1. plot a scatter plot $lg F\left( L \right)\sim lgL$ , and use a first-order least squares fit in two segments, and calculate the slope of the curve:

$\alpha1$ reflects the short-term correlation of HRV, and $\alpha2$ reflects the long-range correlation of HRV.

## Approximate Entropy (ApEn)

1. For a given time sequence $u\left( i \right):1\leq i\leq N$ and given similar tolerance $r$ and embedding dimension $m$, reconstruct to obtain $(N-m+1)$ subsequences $X\left( i \right)=[u\left( i \right), u\left( i+1 \right), u\left( i+2 \right), \ldots, u\left( i+m-1 \right)]$.
2. Calculate the distance $d_{m}[X\left( i \right), X\left( j \right)]$ between any two reconstructed subsequences. The distance is determined by the maximum difference between the corresponding position elements of the two sequences, including the distance when $i = j$.
3. Calculate the ratio $C_{i}^{m}(r)$of the number of distances less than $r$:
4. Calculate the average similarity rates $\Phi_{m}(r)$ and $\Phi_{m+1}(r)$ when the number of subsequences are $m$ and $m+1$:
5. Approximate entropy:

## Sample Entropy (SampEn)

The initial calculation process is the same as ApEn. SampEn is calculated as:

## Fuzzy Entropy (FuzzyEn)

1. Different from step 1 in ApEn, get $X\left( i \right)=[u\left( i \right), u\left( i+1 \right), u\left( i+2 \right), \ldots, u\left( i+m-1 \right)-u_{0}\left( i \right)]$.
2. Calculate the distance $d_{ij}^{m}[X\left( i \right), X\left( j \right)](i\neq j)$ between any two reconstructed subsequences.
3. Introduce fuzzy membership function $A_{ij}^{m}(r)$ and calculate the average $C_{i}^{m}(r)$ for each $i$:
4. Calculate the average similarity rates $\Phi_{m}(r)$ and $\Phi_{m+1}(r)$ when the number of subsequences are $m$ and $m+1$:
5. Fuzzy entropy:

## Multi-Scale Entropy (MSE) and Multi-Scale Fuzzy Entropy (MFE)

1. Time series coarse graining: for the given time series $u\left( i \right):1\leq i\leq N$, construct a continuous coarse-grained time series $y^{\tau}$:

where $\tau$ is the scale factor, as $\tau$ = 1, 2, 3...

1. Calculate the SampEn of each coarse-grained time series $y^{\tau}$.
2. Draw the graph line with the scale factor $\tau$ as the independent variable and the SampEn of the corresponding scale sequence as the dependent variable.

We constructed 25 coarse-grained time series from the original RR time series, in which we obtained 28 indices, including the SampEn value at each coarse-graining scale and 3 complexity metrics, MSEsum5, MSEsum10 and MSEsum20 (defined as the area of the MSE curve at scales 1-5, 1-10 and 1-20, respectively). Because the MSE curve usually rises rapidly at lower scales, peaks at scale 5, and then declines slowly, reaching a plateau after scale 20, calculating the area of the MSE curve at scales 1-5 and 1-20 can portray the complexity characteristics of this curve at high and low resolutions.

Similarly, multi-scale fuzzy entropy (MFE) can be calculated.

## Clinical Applications of Non-linear HRV Analysis

The DFA metrics distinguishes non-linear fluctuations in heart rate time series better than time- and frequency-domain metrics, show correlations with aging and sleep behaviors, and appear to be a significant prognostic indicator that may complement traditional HRV measures in assessing risk, especially in congestive heart failure (Ho et al., 1997; Pikkujämsä et al., 1999; Bunde et al., 2000; Peng et al., 2000; Antonio et al., 2014).

ApEn and SampEn are sensitive to diseases like sepsis, and the metrics respond even before the appearance of clinical signs (Pincus et al., 1991; Lake et al., 2002; Ahmad et al., 2009). SampEn presents high degrees of accuracy in distinguishing atrial fibrillation from normal sinus rhythm (Lake and Moorman, 2011). MSE robustly separates healthy and pathologic groups, differentiates sleep stages (N1, N2 and N3) significantly, and demonstrates a loss of complexity with aging, with an erratic cardiac arrhythmia (atrial fibrillation) and with a life-threatening syndrome (congestive heart failure) (Costa et al., 2002; 2005; Ahmad et al., 2009; Xiao et al., 2012). However, the entropies might also introduce biases, which are thought to be the consequence of the non-stationarity of the datasets and intrinsic limitations of the measure in the context of very low overall variability (Ho et al., 1997).

# Significance of HRV Metrics during Sleep

**Supplementary Table S1.** Correlation coefficients between HRV metrics during different recording period and clinical indicators. Values in the table indicate the level of correlation, *p<0.05，**p<0.01. ^s^ represents Spearman correlation coefficient, and ^p^ represents Pearson correlation coefficient.

| **HRV**  **Metabolic** | **HR-mean** | **SDNN** | **RMSSD** | **SDSD** | **SDANN** | **pNN50** | **TP** | **VLF** | **LF** | **HF** | **LF/HF** | **LFP** | **HFP** | **ApEn** | **SampEn** | **MSE complexity** | **FuzzyEn** | **MFE complexity** | **DFA-α1** | **DFA-α2** |
| --- | --- | --- | --- | --- | --- | --- | --- | --- | --- | --- | --- | --- | --- | --- | --- | --- | --- | --- | --- | --- |
| 24-h | | | | | | | | | | | | | | | | | | | | |
| **Admission FBG** |  |  |  |  |  |  |  |  | 0.406  *^s^ |  |  | 0.468  **^s^ |  |  |  | 0.382±0.027  *^p^ |  | 0.390±0.004  *^p^ |  | -0.507  **^s^ |
| **Discharge FBG** |  |  |  |  |  |  |  |  | -0.408  *^s^ |  |  | -0.380  *^s^ |  |  |  |  |  |  |  |  |
| **HbA1c** |  |  |  |  |  |  | -0.410  *^p^ |  |  |  |  |  |  |  |  |  |  |  |  |  |
| **DBP** |  | 0.402  *^s^ | 0.339  *^s^ | 0.339  *^s^ | 0.409  *^p^ | 0.344  *^s^ |  | 0.359  *^s^ | 0.474  **^s^ | 0.459  **^s^ |  |  | 0.324  *^p^ |  |  |  |  |  |  | -0.342  *^s^ |
| **N%** |  |  |  |  |  |  |  |  |  |  |  | -0.380  *^s^ |  |  |  |  |  |  |  |  |
| **Hb** |  | 0.370  *^s^ | 0.335  *^s^ | 0.335  *^s^ | 0.351  *^p^ | 0.324  *^s^ |  |  | 0.471  **^s^ | 0.412  *^s^ |  | 0.493  **^s^ |  |  |  |  |  |  |  | -0.545  **^s^ |
| **PLT** |  |  |  |  |  | 0.351  *^s^ | -0.394  *^p^ |  | 0.322  *^s^ |  |  |  |  |  |  |  |  |  |  | -0.421  **^s^ |
| **CRP** |  |  |  |  |  |  |  |  |  |  |  |  |  |  |  |  |  |  | -0.359  *^s^ |  |
| **ALT** | 0.331  *^s^ | 0.470  **^s^ | 0.602  **^s^ | 0.602  **^s^ | 0.381  *^s^ | 0.545  **^s^ |  | 0.535  **^s^ | 0.658  **^s^ | 0.688  **^s^ |  |  | 0.466  **^s^ |  |  | -0.352  *^s^ |  | -0.335  *^s^ |  | -0.519  **^s^ |
| **AST** |  |  | 0.338  *^s^ | 0.338  *^s^ |  | 0.349  *^s^ |  | 0.334  *^s^ | 0.457  **^s^ | 0.488  **^s^ |  |  |  |  |  |  |  |  |  | -0.436  **^s^ |
| **AST/ALT** | -0.325  *^s^ | -0.392  *^s^ | -0.572  **^s^ | -0.572  **^s^ | -0.327  *^p^ | -0.650  **^s^ |  | -0.594  **^s^ | -0.707  **^s^ | -0.721  **^s^ |  |  | -0.510  **^s^ |  |  | -0.346  *^s^ |  | -0.338  *^s^ |  | 0.531  **^s^ |
| **GGT** | 0.347  *^s^ |  |  |  |  |  |  |  | 0.350  *^s^ | 0.352  *^s^ |  |  |  |  |  |  |  |  |  | -0.341  *^s^ |
| **BUN** | *p | -0.372  *^s^ | -0.482  **^s^ | -0.482  **^s^ |  | -0.527  **^s^ |  |  | -0.465  **^s^ | -0.517  **^s^ |  |  | -0.459  **^p^ |  |  | -0.353  *^p^ |  | -0.365  *^p^ |  | 0.430  **^s^ |
| **UA** |  |  |  |  |  |  |  |  |  |  |  |  | 0.382  *^p^ |  |  | 0.351  *^p^ |  |  |  |  |
| **TG** |  |  |  |  |  |  |  |  |  |  |  |  |  |  |  |  |  |  |  |  |
| **HDL-C** |  |  |  |  |  |  |  | -0.375  *^s^ | -0.396  *^s^ |  |  |  |  |  |  |  |  |  |  |  |
| **LDL-C** |  |  |  |  |  |  |  |  |  |  |  |  |  |  | 0.364  *^p^ |  | 0.396  *^p^ | 0.369±0.021  *^p^ |  |  |
| **UCr** |  |  |  |  |  |  |  |  | 0.404  *^s^ |  |  | 0.389  *^s^ |  |  |  |  |  |  |  |  |
| **UACR** |  |  |  |  |  |  |  |  |  |  |  | -0.433  *^s^ |  |  | -0.458  *^s^ | -0.492±0.005  **^s^ | -0.361  *^s^ | -0.478±0.008  **^s^ | -0.369  *^s^ |  |
| Sleep | | | | | | | | | | | | | | | | | | | | |
| **Admission FBG** |  |  |  |  |  |  |  |  | 0.389  *^s^ |  |  | 0.491  **^s^ |  |  | 0.362  *^p^ | 0.413±0.034  *^p^ | 0.345  *^p^ | 0.414±0.010  *^s^ |  | -0.575  **^p^ |
| **HbA1c** |  |  |  |  |  |  |  |  |  |  |  |  |  |  |  | 0.422±0.020  *^p^ |  |  |  |  |
| **DBP** |  |  | 0.383  *^s^ | 0.383  *^s^ |  | 0.383  *^s^ |  | 0.330  *^s^ | 0.414  **^s^ | 0.483  **^s^ |  |  |  |  | 0.540  **^p^ | 0.571±0.054  **^p^ | 0.528  **^p^ | 0.537±0.015  **^s^ |  | -0.458  **^p^ |
| **WBC** |  |  |  |  |  |  |  |  |  |  |  |  |  |  |  |  |  |  | -0.376  *^s^ |  |
| **N%** |  |  |  |  |  |  |  |  |  |  |  | -0.361  *^s^ |  |  |  |  |  |  |  |  |
| **Hb** |  |  | 0.430  **^s^ | 0.430  **^s^ |  | 0.418  **^s^ |  |  | 0.505  **^s^ | 0.430  **^s^ |  | 0.321  *^s^ |  | 0.363  *^s^ | 0.491  **^p^ | 0.513±0.031  **^p^ | 0.495  **^p^ | 0.472  **^s^ |  | -0.524  **^p^ |
| **PLT** |  |  | 0.361  *^s^ | 0.361  *^s^ |  | 0.384  *^s^ |  |  | 0.395  *^s^ |  |  |  |  |  |  |  |  |  |  | -0.381  *^p^ |
| **CRP** |  |  |  |  |  |  |  |  |  |  |  |  |  |  |  |  |  |  | -0.399  **^s^ |  |
| **ALT** |  |  | 0.629  **^s^ | 0.629  **^s^ |  | 0.573  **^s^ |  | 0.494  **^s^ | 0.624  **^s^ | 0.638  **^s^ |  |  | 0.449  **^s^ |  | 0.645  **^s^ | 0.574±0.032  **^s^ | 0.645  **^s^ | 0.619±0.028  **^s^ |  | -0.435  **^s^ |
| **AST** |  |  | 0.440  **^s^ | 0.440  **^s^ |  | 0.399  *^s^ |  |  | 0.449  **^s^ | 0.434  **^s^ |  |  |  | 0.357  *^s^ | 0.487  **^s^ | 0.449±0.019  **^s^ | 0.449  **^s^ | 0.447±0.001  *^s^ |  | -0.336  *^s^ |
| **AST/ALT** |  |  | -0.692  **^s^ | -0.692  **^s^ |  | -0.657  **^s^ |  | -0.553  **^s^ | -0.660  **^s^ | -0.691  **^s^ |  |  | -0.496  **^s^ |  | -0.641  **^s^ | -0.593±0.045  **^s^ | -0.693  **^s^ | -0.711±0.021  **^s^ |  | 0.500  **^s^ |
| **GGT** |  |  | 0.331  *^s^ | 0.331  *^s^ |  |  |  |  |  | 0.325  *^s^ |  |  |  |  | 0.489  **^s^ | 0.492±0.011  **^s^ | 0.407  *^s^ | 0.454±0.021  **^s^ |  | -0.331  *^s^ |
| **BUN** |  |  | -0.535  **^s^ | -0.535  **^s^ |  | -0.556  **^s^ |  |  | -0.436  *^s^ | -0.522  **^s^ | 0.358  *^s^ |  | -0.409  *^p^ |  | -0.541  **^p^ | -0.525  **^p^ | -0.521  **^p^ | -0.533±0.041  **^s^ |  |  |
| **UA** |  |  |  |  |  |  |  |  |  |  |  |  |  |  | 0.435  *^p^ |  | 0.413  *^p^ |  |  |  |
| **TG** |  |  |  |  | 0.351  *^s^ |  |  |  |  |  |  |  |  |  | 0.448  **^s^ | 0.397±0.021  *^s^ |  |  |  |  |
| **LDL-C** |  |  |  |  |  |  |  |  |  |  |  |  |  |  | 0.474  **^p^ |  | 0.440  *^p^ |  |  |  |
| **UMA** |  |  |  |  |  |  |  |  |  |  |  |  |  | -0.380  *^s^ |  |  |  |  |  |  |
| **UCr** |  |  |  |  |  |  |  |  |  | 0.358  *^s^ |  |  |  |  |  |  |  |  |  |  |
| **UACR** |  |  |  |  |  |  |  |  |  |  |  |  |  | -0.504  **^s^ |  | -0.400±0.013  **^s^ | -0.512  **^s^ |  |  |  |
| Awake | | | | | | | | | | | | | | | | | | | | |
| **Admission FBG** |  |  |  |  |  |  |  |  | 0.362  *^s^ |  | 0.355  *^p^ | 0.506  **^s^ |  |  |  |  |  | 0.369±0.026  *^s^ | 0.389  *^p^ | -0.452  **^s^ |
| **Discharge FBG** |  |  |  |  |  |  |  |  | -0.417  *^s^ |  |  | -0.390  *^s^ |  |  |  |  |  |  |  |  |
| **HbA1c** |  |  |  |  |  |  | -0.518  **^s^ |  |  |  |  | 0.415  *^s^ |  |  |  |  |  |  | 0.391  *^p^ |  |
| **DBP** | 0.404  *^p^ |  |  |  |  |  |  |  | 0.425  **^s^ | 0.341  *^s^ |  | 0.351  *^s^ |  |  |  |  |  |  |  | -0.377  *^s^ |
| **N%** |  |  |  |  |  |  |  |  |  |  |  | -0.411  **^s^ |  |  |  |  |  |  |  | 0.377  *^s^ |
| **Hb** |  |  |  |  |  |  |  |  | 0.438  **^s^ | 0.330  *^s^ |  | 0.636  **^s^ |  |  |  | 0.357±0.022  *^p^ |  |  | 0.399  *^p^ | -0.662  **^s^ |
| **PLT** |  |  |  |  |  |  | -0.363  *^s^ |  |  |  |  |  |  |  |  |  |  |  |  |  |
| **ALT** | 0.375  **^s^ |  | 0.522  **^s^ | 0.522  **^s^ |  | 0.498  **^s^ |  | 0.461  **^s^ | 0.632  **^s^ | 0.628  **^s^ |  | 0.407  **^s^ | 0.523  **^s^ | 0.362  *^s^ |  | 0.333  *^s^ |  | 0.338±0.004  *^s^ |  | -0.540  **^s^ |
| **AST** |  |  | 0.398  *^s^ | 0.398  *^s^ |  | 0.337  *^s^ |  | 0.362  *^s^ | 0.486  **^s^ | 0.461  **^s^ |  | 0.331  *^s^ | 0.355  *^s^ |  |  |  |  |  |  | -0.439  *^s^ |
| **AST/ALT** |  |  | -0.579  **^s^ | -0.579  **^s^ |  | -0.583  **^s^ |  | -0.426  **^s^ | -0.592  **^s^ | -0.606  **^s^ |  | -0.376  *^s^ | -0.539  **^s^ | -0.334  *^s^ |  |  |  | -0.329  *^s^ |  | 0.554  **^s^ |
| **GGT** | 0.371  *^s^ |  |  |  |  |  |  |  |  |  |  |  |  |  |  |  |  |  |  |  |
| **BUN** | -0.363  *^p^ |  | -0.441  *^s^ | -0.441  *^s^ |  | -0.491  **^s^ |  |  | -0.455  *^s^ | -0.466  **^s^ |  | -0.372  *^s^ | -0.431  *^p^ |  |  | -0.403±0.028  *^p^ |  | -0.404±0.029  *^s^ |  | 0.496  **^s^ |
| **UA** |  |  |  |  |  |  |  |  |  |  |  |  | 0.420  *^p^ |  |  |  |  |  |  |  |
| **TG** |  |  |  |  |  |  |  |  |  |  |  |  | 0.350  *^s^ |  |  |  |  |  |  |  |
| **HDL-C** |  |  |  |  |  |  |  |  |  |  |  |  |  |  |  |  |  |  |  |  |
| **LDL-C** |  |  |  |  |  |  |  |  |  |  |  |  |  |  |  |  |  | 0.378±0.019  *^p^ |  |  |
| **UCr** |  |  |  |  |  |  |  |  | 0.441  *^s^ |  |  | 0.423  *^s^ |  | 0.356  *^p^ |  |  |  |  |  | -0.351  *^s^ |
| **UACR** |  |  |  |  |  |  |  |  |  |  |  |  |  |  |  | -0.358  *^s^ |  |  | -0.403  *^s^ |  |

# HRV Metrics in Different Sleep Stages are More Associated with T2DM Clinical Indicators

**Supplementary Table S2.** The HRV metrics of the subjects in three sleep stages. Data are presented as mean ± SD if the variable is normally distributed, otherwise presented as median (p25, p75). ^a^ represents a significant difference between unstable sleep and stable sleep stages, ^b^ represents a significant difference between stable sleep and REM sleep stages, and ^c^ represents a significant difference between unstable sleep and REM sleep stages, p<0.05.

| **Metric (Unit)** | **Unstable Sleep** | **Stable Sleep** | **REM Sleep** | **p-value** |
| --- | --- | --- | --- | --- |
| HR-mean (bpm) | 69.49±8.24 | 66.26±8.23 | 70.97±8.62 | **0.047^b^** |
| SDNN (ms) | 89.22±31.67 | 57.38 (36.88, 72.11) | 89.24±31.14 | **<0.001^ab^** |
| RMSSD (ms) | 23.76 (16.41, 39.24) | 25.12 (16.88, 41.59) | 21.05 (15.60, 36.13) | 0.770 |
| SDSD (ms) | 23.76 (16.41, 39.24) | 25.13 (16.88, 41.60) | 21.05 (15.60, 36.13) | 0.770 |
| SDANN (ms) | 50.35 (37.73, 68.98) | 39.28 (26.33, 50.07) | 55.92 (41.43, 68.71) | **0.003^ab^** |
| pNN50 (%) | 3.34 (0.61, 16.70) | 3.32 (0.26, 17.88) | 2.42 (0.51, 13.47) | 0.948 |
| TP (×10^6^ ms^2^) | 5.62 (3.86, 9.60) | 4.79 (2.43, 7.68) | 2.47 (1.71, 4.01) | **<0.001^bc^** |
| VLF (×10^3^ ms^2^) | 37.51 (17.88, 63.58) | 5.94 (3.34, 18.03) | 17.16 (7.45, 34.11) | **<0.001^abc^** |
| LF (×10^3^ ms^2^) | 7.20 (2.53, 14.68) | 2.66 (0.89, 5.10) | 2.73 (0.90, 5.31) | **<0.001^ac^** |
| HF (×10^2^ ms^2^) | 20.89 (11.12, 39.49) | 19.86 (6.37, 58.41) | 7.72 (2.82, 24.90) | **0.020^c^** |
| LF/HF | 3.22 (2.27, 5.04) | 1.34 (0.69, 2.13) | 2.77 (1.95, 4.30) | **<0.001^ab^** |
| LFP (%) | 32.63 (32.03, 33.50) | 32.15 (30.76, 32.86) | 32.19 (31.42, 32.84) | **0.042^a^** |
| HFP (%) | 28.37±2.27 | 31.03±2.92 | 27.60±2.63 | **<0.001^ab^** |
| DFA-α1 | 1.37 (1.19, 1.47) | 0.97±0.22 | 1.27±0.24 | **<0.001^ab^** |
| DFA-α2 | 1.21±0.12 | 1.05±0.14 | 1.26±0.11 | **<0.001^ab^** |
| ApEn | 1.85 (1.41, 2.08) | 1.87 (1.49, 2.05) | 1.40±0.42 | **<0.001^bc^** |
| SampEn | 1.08±0.31 | 1.60 (1.43, 1.76) | 1.00±0.33 | **<0.001^ab^** |
| FuzzyEn | 1.87±0.44 | 2.17±0.43 | 1.77±0.44 | **<0.001^ab^** |
| MSEsum5 | 5.61±1.46 | 7.65 (6.40, 8.18) | 5.14±1.57 | **<0.001^ab^** |
| MSEsum10 | 12.40±2.92 | 15.18 (13.13, 16.43) | 11.29±3.15 | **<0.001^ab^** |
| MSEsum20 | 26.93±5.62 | 29.54 (25.14, 31.91) | 24.58±6.21 | **0.007^b^** |
| MFEsum5 | 9.71±2.18 | 10.69 (9.65, 11.61) | 9.19±2.30 | **0.031^b^** |
| MFEsum10 | 20.83±4.30 | 21.86 (19.72, 23.61) | 19.72±4.69 | 0.293 |
| MFEsum20 | 44.36±8.25 | 43.09 (38.57, 46.64) | 43.79 (37.26, 47.69) | 0.255 |

**Supplementary Table S3.** Correlation coefficients between HRV metrics in three sleep stages and clinical indicators. Values in the table indicate the level of correlation, *p<0.05，**p<0.01. ^s^ represents Spearman correlation coefficient, and ^p^ represents Pearson correlation coefficient.

1. Correlation coefficients between linear HRV metrics and clinical indicators

| **Linear HRV**  **Metabolic** | **Time-domain** | | | | | **Frequency-domain** | | | | | | |
| --- | --- | --- | --- | --- | --- | --- | --- | --- | --- | --- | --- | --- |
|  | **SDNN** | **RMSSD** | **SDSD** | **SDANN** | **pNN50** | **TP** | **VLF** | **LF** | **HF** | **LF/HF** | **LFP** | **HFP** |
| **Unstable Sleep** | | | | | | | | | | | | |
| **Admission FBG** |  |  |  |  |  |  |  | 0.350  *^s^ |  |  | 0.440  **^s^ |  |
| **DBP** |  | 0.389  *^s^ | 0.389  *^s^ |  | 0.381  *^s^ |  |  | 0.346  *^s^ | 0.408  *^s^ |  |  | 0.323  *^p^ |
| **N%** |  |  |  |  |  |  |  | -0.361  *^s^ |  |  |  |  |
| **Hb** |  | 0.451  **^s^ | 0.451  **^s^ |  | 0.406  *^s^ |  |  | 0.472  **^s^ | 0.433  **^s^ |  | 0.381  *^s^ |  |
| **PLT** |  | 0.359  *^s^ | 0.359  *^s^ |  | 0.367  *^s^ |  |  |  |  |  |  |  |
| **ALT** |  | 0.665  **^s^ | 0.665  **^s^ |  | 0.610  **^s^ |  | 0.436  **^s^ | 0.525  **^s^ | 0.682  **^s^ |  |  | 0.519  **^s^ |
| **AST** |  | 0.477  **^s^ | 0.477  **^s^ |  | 0.423  *^s^ |  | 0.355  *^s^ | 0.410  *^s^ | 0.508  **^s^ |  |  | 0.363  *^s^ |
| **AST/ALT** | -0.424  **^s^ | -0.710  **^s^ | -0.710  **^s^ |  | -0.701  **^s^ |  | -0.416  *^s^ | -0.540  **^s^ | -0.716  **^s^ |  |  | -0.551  **^s^ |
| **GGT** |  | 0.360  *^s^ | 0.360  *^s^ |  | 0.330  *^s^ |  |  | 0.328  *^s^ | 0.326  *^s^ |  |  |  |
| **BUN** |  | -0.516  **^s^ | -0.516  **^s^ |  | -0.533  **^s^ |  |  |  | -0.476  **^s^ |  |  |  |
| **TG** |  |  |  |  |  | -0.494  **^s^ |  |  |  |  |  |  |
| **UCr** |  | 0.381  *^s^ | 0.381  *^s^ |  |  |  |  |  | 0.373  *^s^ |  |  |  |
| **Stable Sleep** | | | | | | | | | | | | |
| **Discharge FBG** |  |  |  |  |  |  |  | -0.425  *^s^ |  |  |  |  |
| **SBP** |  |  |  | 0.365  *^s^ |  |  |  |  |  |  |  |  |
| **DBP** |  | 0.332  *^s^ | 0.332  *^s^ |  |  |  | 0.335  *^s^ | 0.392  *^s^ | 0.450  **^s^ |  |  |  |
| **N%** |  |  |  |  |  |  |  |  |  |  | -0.435  **^s^ |  |
| **Hb** |  | 0.345  *^s^ | 0.345  *^s^ |  |  |  |  | 0.374  *^s^ | 0.363  *^s^ |  |  |  |
| **PLT** |  |  |  |  | 0.353  *^s^ |  |  |  |  |  |  |  |
| **ALT** |  | 0.568  **^s^ | 0.568  **^s^ |  | 0.525  **^s^ |  | 0.383  *^s^ | 0.510  **^s^ | 0.581  **^s^ | -0.387  *^s^ |  | 0.489  **^s^ |
| **AST** |  | 0.403  *^s^ | 0.403  *^s^ |  | 0.367  *^s^ |  |  | 0.368  *^s^ | 0.410  *^s^ |  |  |  |
| **AST/ALT** | -0.362  *^s^ | -0.654  **^s^ | -0.654  **^s^ |  | -0.620  **^s^ |  | -0.428  **^s^ | -0.554  **^s^ | -0.623  **^s^ | 0.476  **^s^ |  | -0.556  **^s^ |
| **GGT** |  |  |  |  | 0.335  *^s^ |  |  |  | 0.375  *^s^ |  |  |  |
| **BUN** |  | -0.506  **^s^ | -0.506  **^s^ |  | -0.493  **^s^ |  |  | -0.478  **^s^ | -0.524  **^s^ | 0.393  *^s^ |  | -0.414  *^s^ |
| **TG** |  |  |  |  |  |  |  |  |  |  |  | 0.354  *^s^ |
| **UCr** |  |  |  |  |  |  |  |  |  |  |  | 0.393  *^s^ |
| **UACR** |  |  |  |  |  |  |  |  |  |  | -0.471  **^s^ |  |
| **REM Sleep** | | | | | | | | | | | | |
| **Admission FBG** |  | 0.350  *^s^ | 0.350  *^s^ |  |  |  |  |  |  |  | 0.539  **^s^ |  |
| **DBP** |  | 0.354  *^s^ | 0.354  *^s^ |  | 0.336  *^s^ |  |  | 0.332  *^s^ | 0.424  **^s^ |  |  | 0.372  *^p^ |
| **Hb** |  | 0.410  *^s^ | 0.410  *^s^ |  | 0.362  *^s^ |  | 0.375  *^s^ | 0.510  **^s^ | 0.478  *^s^ |  | 0.406  *^s^ | 0.390  *^p^ |
| **PLT** |  | 0.380  *^s^ | 0.380  *^s^ |  | 0.369  *^s^ |  |  | 0.328  *^s^ |  |  |  |  |
| **CRP** |  |  |  |  |  |  |  |  |  | -0.340  *^s^ |  |  |
| **ALT** |  | 0.586  **^s^ | 0.586  **^s^ |  | 0.552  **^s^ |  | 0.340  *^s^ | 0.475  **^s^ | 0.490  *^s^ |  |  | 0.430  **^s^ |
| **AST** |  | 0.387  *^s^ | 0.387  *^s^ |  | 0.339  *^s^ |  |  | 0.376  *^s^ |  |  |  |  |
| **AST/ALT** | -0.346  *^s^ | -0.680  **^s^ | -0.680  **^s^ |  | -0.699  **^s^ |  | -0.405  *^s^ | -0.489  **^s^ | -0.561  **^s^ |  |  | -0.509  **^s^ |
| **GGT** |  |  |  | -0.341  *^s^ |  |  |  |  |  |  |  | 0.377  *^s^ |
| **BUN** |  | -0.507  **^s^ | -0.507  **^s^ |  | -0.529  **^s^ |  |  | -0.410  *^s^ | -0.524  **^s^ | 0.372  *^s^ |  | -0.483  **^p^ |
| **UCr** |  | 0.388  *^s^ | 0.388  *^s^ |  |  |  |  | 0.366  *^s^ | 0.433  *^s^ |  |  | 0.382  *^p^ |

1. Correlation coefficients between non-linear HRV metrics and clinical indicators

| **Non-linear HRV**  **Metabolic** | **DFA-α1** | **DFA-α2** | **ApEn** | **SampEn** | **MSE** | **MSE complexity** | **FuzzyEn** | **MFE** | **MFE complexity** |
| --- | --- | --- | --- | --- | --- | --- | --- | --- | --- |
| Unstable Sleep | | | | | | | | | |
| **Admission FBG** |  | -0.584  **^p^ |  | 0.400  *^p^ | 0.466  **^s^ | 0.440  **^p^ | 0.378  *^p^ | 0.436  *^s^ | 0.403±0.003  *^p^ |
| **HbA1c** |  | -0.412  *^p^ |  | 0.438  *^p^ | 0.411±0.016  *^s^ | 0.409±0.017  *^p^ |  |  |  |
| **DBP** |  |  |  | 0.523  **^p^ | 0.488±0.047  **^s^ | 0.545±0.060  **^p^ |  | 0.457±0.043  **^s^ | 0.543±0.039  **^p^ |
| **WBC** | -0.396  *^s^ |  |  |  |  |  |  |  |  |
| **N%** |  |  |  |  |  | -0.339  *^p^ |  | -0.429  **^s^ |  |
| **Hb** |  | -0.496  **^p^ |  | 0.483  **^p^ | 0.424±0.001  **^s^ | 0.494±0.031  **^p^ | 0.500  **^p^ | 0.435±0.009  **^s^ | 0.490±0.051  **^p^ |
| **PLT** |  |  |  |  |  |  |  |  |  |
| **CRP** | -0.356  *^s^ |  |  |  |  |  |  |  |  |
| **ALT** |  | -0.396  *^s^ |  | 0.572  **^s^ | 0.472±0.047  **^s^ | 0.520±0.029  **^p^ | 0.615  **^s^ | 0.538±0.048  **^s^ | 0.590±0.012  **^s^ |
| **AST** |  |  |  | 0.397  *^s^ | 0.426  **^s^ | 0.385±0.018  *^p^ | 0.440  **^s^ | 0.461±0.043  **^s^ | 0.439  **^s^ |
| **AST/ALT** |  | 0.421  *^s^ |  | -0.614  **^s^ | -0.503±0.063  **^s^ | -0.583±0.040  **^p^ | -0.656  **^s^ | -0.614±0.052  **^s^ | -0.680±0.014  **^s^ |
| **GGT** |  |  |  | 0.345  *^s^ | 0.455±0.034  **^s^ | 0.452  **^p^ | 0.329  *^s^ | 0.478±0.048  **^s^ | 0.459  **^s^ |
| **BUN** |  |  |  | -0.443  *^p^ | -0.400±0.035  *^s^ | -0.408±0.044  *^p^ | -0.451  *^p^ | -0.488±0.032  **^s^ | -0.463  **^p^ |
| **UA** |  |  |  | 0.379  *^p^ | 0.351  *^s^ |  | 0.356  *^p^ | 0.353  *^s^ |  |
| **TG** |  |  |  | 0.405  *^s^ | 0.361±0.022  *^s^ | 0.361±0.011  *^p^ |  |  |  |
| **HDL-C** |  |  |  |  |  |  |  | -0.345  *^s^ |  |
| **LDL-C** |  |  |  | 0.460  **^p^ | 0.386  *^s^ |  | 0.428  *^s^ | 0.372  *^s^ |  |
| **UCr** |  | -0.375  *^p^ |  |  |  |  | 0.361  *^p^ |  | 0.371±0.016  **^p^ |
| **UACR** |  |  | -0.365  *^s^ |  | -0.381±0.009  **^s^ |  |  |  |  |
| Stable Sleep | | | | | | | | | |
| **Admission FBG** |  |  |  | 0.362  *^s^ | 0.353±0.015  *^s^ | 0.364  *^s^ |  | 0.377  *^s^ |  |
| **Discharge FBG** |  |  |  |  | -0.423  *^s^ |  |  |  |  |
| **HbA1c** |  |  |  |  | 0.389  *^s^ |  |  |  |  |
| **SBP** |  |  |  |  |  |  |  | 0.442  **^s^ |  |
| **DBP** |  |  |  | 0.458  **^s^ | 0.463±0.039  **^s^ | 0.447  **^s^ | 0.508  **^p^ | 0.456±0.033  **^s^ | 0.474±0.027  **^s^ |
| **WBC** | -0.456  **^s^ |  |  |  |  |  |  |  |  |
| **N%** | 0.448  **^p^ |  |  |  | -0.462±0.031  **^s^ | -0.461±0.035  *^s^ |  | -0.474  **^s^ |  |
| **Hb** |  | -0.535  **^p^ |  | 0.498  **^s^ | 0.469±0.037  **^s^ | 0.476±0.070  *^s^ | 0.475  **^p^ | 0.380±0.026  *^s^ | 0.431  **^s^ |
| **PLT** |  |  |  | 0.364  *^s^ | 0.364  *^s^ |  |  | 0.346  *^s^ |  |
| **CRP** | -0.442  **^s^ |  |  |  | -0.347  *^s^ |  |  | -0.375±0.029  **^s^ |  |
| **ALT** | -0.381  *^p^ | -0.419  *^s^ |  | 0.786  **^s^ | 0.553±0.114  **^s^ | 0.596±0.076  **^s^ | 0.721  **^s^ | 0.580±0.070  **^s^ | 0.635±0.042  **^s^ |
| **AST** |  | -0.496  **^s^ |  | 0.630  **^s^ | 0.523±0.058  **^s^ | 0.555±0.051  **^s^ | 0.522  **^s^ | 0.488±0.054  **^s^ | 0.491±0.035  **^s^ |
| **AST/ALT** | 0.425  **^s^ | 0.376  *^s^ |  | -0.728  **^s^ | -0.571±0.132  **^s^ | -0.516±0.095  **^s^ | -0.760  **^s^ | -0.585±0.075  **^s^ | -0.667±0.055  **^s^ |
| **GGT** |  |  |  | 0.441  **^s^ | 0.434±0.011  **^s^ | 0.378±0.024  *^s^ | 0.442  *^s^ | 0.482±0.060  **^s^ | 0.447±0.007  *^s^ |
| **BUN** |  |  |  | -0.625  *^s^ | -0.570±0.078  **^s^ | -0.396±0.057  **^s^ | -0.576  **^p^ | -0.545±0.072  **^s^ | -0.534±0.047  **^s^ |
| **UA** |  |  |  |  |  |  | 0.387  *^p^ | 0.352  *^s^ |  |
| **TG** |  |  |  | 0.386  *^s^ | 0.386  *^s^ |  |  |  |  |
| **LDL-C** |  |  |  |  |  |  | 0.354  *^p^ |  |  |
| **UCr** |  |  |  |  |  |  | 0.370  *^p^ | 0.375  *^s^ |  |
| **UACR** |  | 0.378  *^s^ |  |  | -0.413±0.041  *^s^ |  |  | -0.363  *^s^ |  |
| REM Sleep | | | | | | | | | |
| **Admission FBG** |  | -0.524  **^p^ |  |  | 0.403±0.023  *^s^ |  |  | 0.394±0.024  *^s^ |  |
| **HbA1c** |  |  |  | 0.424  *^p^ | 0.418±0.022  *^s^ | 0.442±0.021  *^p^ |  |  |  |
| **DBP** |  | -0.507  **^p^ |  | 0.493  **^p^ | 0.502±0.070  **^s^ | 0.560±0.046  **^p^ | 0.496  **^p^ | 0.489±0.049  **^s^ | 0.533±0.029  **^s^ |
| **N%** |  |  |  |  | -0.332±0.008  *^s^ |  |  | -0.456±0.045  **^s^ |  |
| **Hb** |  | -0.549  **^p^ |  | 0.475  **^p^ | 0.479±0.037  **^s^ | 0.524±0.043  **^p^ | 0.504  **^p^ | 0.478±0.054  **^s^ | 0.464±0.040  **^s^ |
| **PLT** |  | -0.368  *^p^ |  |  |  |  |  | 0.515  **^s^ |  |
| **CRP** | -0.359  *^s^ |  |  |  |  |  |  |  |  |
| **ALT** |  | -0.402  *^s^ |  | 0.581  **^s^ | 0.512±0.041  **^s^ | 0.552±0.011  **^s^ | 0.591  **^s^ | 0.543±0.061  **^s^ | 0.590±0.007  **^s^ |
| **AST** |  |  |  | 0.416  *^s^ | 0.437±0.019  **^s^ | 0.432±0.003  *^s^ | 0.416  *^s^ | 0.476±0.039  **^s^ | 0.402±0.008  *^s^ |
| **AST/ALT** |  | 0.437  **^s^ | 0.366  *^s^ | -0.575  **^s^ | -0.493±0.042  **^s^ | -0.518±0.029  **^s^ | -0.627  **^s^ | -0.570±0.070  **^s^ | -0.649±0.001  **^s^ |
| **GGT** |  |  |  | 0.478  **^s^ | 0.462±0.023  **^s^ | 0.494±0.009  **^s^ | 0.400  *^s^ | 0.468±0.026  **^s^ | 0.436  **^s^ |
| **BUN** |  | 0.384  *^p^ |  | -0.472  **^p^ | -0.495±0.030  **^s^ | -0.486  **^p^ | -0.496  **^p^ | -0.535±0.043  **^s^ | -0.578±0.010  **^s^ |
| **UA** |  |  |  |  | 0.376  *^s^ |  |  |  |  |
| **TG** |  | -0.418  *^s^ |  |  | 0.357  *^s^ | 0.354  *^s^ |  |  |  |
| **HDL-C** |  |  | 0.404  *^p^ |  |  |  |  |  |  |
| **LDL-C** |  | -0.495  **^p^ |  | 0.422  *^p^ | 0.358±0.008  *^s^ | 0.400±0.010  *^p^ | 0.400  *^p^ | 0.484±0.036  **^s^ | 0.382±0.026  *^s^ |
| **UMA** |  |  |  |  | -0.466  *^s^ |  | 0.344  *^s^ | -0.512±0.008  **^s^ |  |
| **UCr** |  | -0.433  *^p^ |  |  |  |  | 0.358  *^p^ |  |  |
| **UACR** |  |  | -0.405  *^s^ |  | -0.505±0.027  **^s^ | -0.488±0.004  **^s^ |  | -0.514±0.046  **^s^ | -0.404±0.003  *^s^ |

# Correlation Analysis between HRV Metrics in Specific Time Periods and Clinical Indicators of Metabolic Function

In our original study, we also used RR interval time series from four time periods (late night, 3:00-4:00; dawn, 6:00-7:00; after lunch, 13:00-14:00; after dinner, 19:00-20:00) to perform HRV analysis and correlation analysis with clinical indicators.

At dawn (6:00-7:00), the correlation between BUN and HRV metrics was relatively high in all time periods, and the correlations between HRV metrics and clinical indicators including DBP and Hb was similar to those during sleep period, which were weaker during awake period. Correlations between HRV metrics and clinical indicators including HDL-C and LDL-C were mainly found after lunch (13:00-14:00). After dinner (19:00-20:00) the correlations between LF/HF and clinical indicators were found and the correlations between LFP and clinical indicators were elevated.

**Supplementary Table S4.** Spearman correlation coefficients between HRV metrics in late night (3:00-4:00) and clinical indicators. Values in the table indicate the level of correlation and are significant, i.e., p<0.05.

| **HRV**  **Metabolic** | **HR-mean** | **RMSSD** | **pNN50** | **LFP** | **MSEsum5** | **MSEsum20** |
| --- | --- | --- | --- | --- | --- | --- |
| **Admission FBG** |  |  | 0.30 |  |  |  |
| **DBP** |  | 0.34 | 0.31 | 0.30 |  |  |
| **N%** |  |  |  | -0.45 |  |  |
| **Hb** |  |  |  | 0.45 | 0.42 | 0.40 |
| **ALT** |  | 0.37 | 0.31 |  | 0.43 | 0.42 |
| **GGT** | 0.40 |  |  |  |  |  |
| **BUN** |  | -0.36 | -0.34 |  | -0.52 | -0.49 |
| **UCr** |  |  |  | 0.37 | 0.41 | 0.43 |

**Supplementary Table S5.** Spearman correlation coefficients between HRV metrics in dawn (6:00-7:00) and clinical indicators. Values in the table indicate the level of correlation and are significant, i.e., p<0.05.

| **HRV**  **Metabolic** | **RMSSD** | **pNN50** | **LFP** | **HFP** | **SampEn** | **MSEsum5** | **MSEsum20** |
| --- | --- | --- | --- | --- | --- | --- | --- |
| **Admission FBG** |  |  | 0.42 |  |  |  |  |
| **DBP** | 0.31 |  | 0.38 | 0.37 |  | 0.36 |  |
| **Hb** | 0.39 | 0.37 | 0.41 | 0.33 | 0.46 | 0.32 |  |
| **CRP** |  |  |  |  |  | -0.39 | -0.39 |
| **ALT** | 0.39 | 0.39 | 0.36 | 0.35 | 0.37 |  |  |
| **GGT** |  |  |  |  | 0.33 |  |  |
| **BUN** | -0.55 | -0.54 |  | -0.57 | -0.50 |  |  |
| **HDL-C** | -0.35 | -0.34 |  |  |  |  |  |
| **LDL-C** |  |  | 0.45 |  |  |  |  |

**Supplementary Table S6.** Spearman correlation coefficients between HRV metrics after lunch (13:00-14:00) and clinical indicators. Values in the table indicate the level of correlation and are significant, i.e., p<0.05.

| **HRV**  **Metabolic** | **HR-mean** | **RMSSD** | **pNN50** | **LF/HF** | **LFP** | **HFP** | **MSEsum5** | **MSEsum20** |
| --- | --- | --- | --- | --- | --- | --- | --- | --- |
| **Admission FBG** |  |  |  |  | 0.35 |  |  |  |
| **Discharge FBG** |  |  |  |  | -0.51 |  | -0.39 |  |
| **DBP** | 0.36 |  |  |  | 0.40 |  | 0.31 |  |
| **N%** | -0.39 |  |  |  |  |  |  |  |
| **Hb** | 0.39 |  |  |  | 0.54 |  | 0.38 | 0.33 |
| **ALT** | 0.37 |  |  |  | 0.50 |  | 0.38 | 0.36 |
| **GGT** | 0.37 |  |  |  |  |  |  |  |
| **BUN** |  | -0.41 | -0.45 |  | -0.58 | -0.41 | -0.38 | -0.43 |
| **UA** |  |  |  |  |  | 0.43 |  |  |
| **HDL-C** |  |  |  |  | -0.55 |  |  |  |
| **LDL-C** |  | 0.40 | 0.50 |  |  | 0.47 |  | 0.34 |
| **UCr** |  |  |  | 0.42 | 0.48 |  |  |  |

**Supplementary Table S7.** Spearman correlation coefficients between HRV metrics after dinner (19:00-20:00) and clinical indicators. Values in the table indicate the level of correlation and are significant, i.e., p<0.05.

| **HRV**  **Metabolic** | **HR-mean** | **RMSSD** | **pNN50** | **LF/HF** | **LFP** | **HFP** | **MSEsum5** | **MSEsum20** |
| --- | --- | --- | --- | --- | --- | --- | --- | --- |
| **Admission FBG** |  |  |  | 0.41 | 0.50 |  |  |  |
| **HbA1c** | 0.56 |  |  |  |  |  |  |  |
| **DBP** | 0.38 |  |  |  |  |  | 0.37 |  |
| **N%** | -0.31 | 0.31 | 0.33 | -0.41 | -0.45 |  |  |  |
| **Hb** | 0.36 |  |  | 0.38 | 0.63 |  |  |  |
| **ALT** | 0.40 |  |  |  | 0.44 |  | 0.48 | 0.36 |
| **GGT** | 0.41 |  |  |  |  |  | 0.58 | 0.47 |
| **BUN** | -0.38 |  |  |  | -0.44 |  | -0.40 |  |
| **UA** |  |  |  |  |  | 0.34 | 0.44 | 0.40 |
| **UCr** |  |  |  |  | 0.43 |  |  |  |
| **UACR** |  |  |  | -0.35 | -0.48 |  |  |  |

# References

Ahmad, S., Ramsay, T., Huebsch, L., Flanagan, S., McDiarmid, S., Batkin, I., et al. (2009). Continuous Multi-Parameter Heart Rate Variability Analysis Heralds Onset of Sepsis in Adults. *PLOS ONE* 4(8)**,** e6642. doi: 10.1371/journal.pone.0006642.

Antonio, A.M., Cardoso, M.A., Abreu, L.C.d., Raimundo, R.D., Fontes, A.M.G., Silva, A.G.d., et al. (Year). "Fractal Dynamics of Heart Rate Variability: A Study in Healthy Subjects").

Bunde, A., Havlin, S., Kantelhardt, J.W., Penzel, T., Peter, J.-H., and Voigt, K. (2000). Correlated and Uncorrelated Regions in Heart-Rate Fluctuations during Sleep. *Physical Review Letters* 85(17)**,** 3736-3739. doi: 10.1103/PhysRevLett.85.3736.

Costa, M., Goldberger, A.L., and Peng, C.K. (2002). Multiscale Entropy Analysis of Complex Physiologic Time Series. *Physical Review Letters* 89(6)**,** 068102. doi: 10.1103/PhysRevLett.89.068102.

Costa, M., Goldberger, A.L., and Peng, C.K. (2005). Multiscale entropy analysis of biological signals. *Physical Review E* 71(2)**,** 021906. doi: 10.1103/PhysRevE.71.021906.

Goldberger, A.L., and West, B.J. (1987). Applications of Nonlinear Dynamics to Clinical Cardiology. Annals of the New York Academy of Sciences 504(1), 195-213. doi: https://doi.org/10.1111/j.1749-6632.1987.tb48733.x.

Ho, K.K.L., Moody, G.B., Peng, C.-K., Mietus, J.E., Larson, M.G., Levy, D., et al. (1997). Predicting Survival in Heart Failure Case and Control Subjects by Use of Fully Automated Methods for Deriving Nonlinear and Conventional Indices of Heart Rate Dynamics. *Circulation* 96(3)**,** 842-848. doi: doi:10.1161/01.CIR.96.3.842.

Lake, D.E., and Moorman, J.R. (2011). Accurate estimation of entropy in very short physiological time series: the problem of atrial fibrillation detection in implanted ventricular devices. *American Journal of Physiology-Heart and Circulatory Physiology* 300(1)**,** H319-H325. doi: 10.1152/ajpheart.00561.2010.

Lake, D.E., Richman, J.S., Griffin, M.P., and Moorman, J.R. (2002). Sample entropy analysis of neonatal heart rate variability. *American Journal of Physiology-Regulatory, Integrative and Comparative Physiology* 283(3)**,** R789-R797. doi: 10.1152/ajpregu.00069.2002.

Peng, C.-k., Hausdorff, J.M., and Goldberger, A.L. (2000). "Fractal mechanisms in neuronal control: human heartbeat and gait dynamics in health and disease," in *Self-Organized Biological Dynamics and Nonlinear Control: Toward Understanding Complexity, Chaos and Emergent Function in Living Systems,* ed. J. Walleczek. (Cambridge: Cambridge University Press), 66-96.

Pikkujämsä, S.M., Mäkikallio, T.H., Sourander, L.B., Räihä, I.J., Puukka, P., Skyttä, J., et al. (1999). Cardiac Interbeat Interval Dynamics From Childhood to Senescence. *Circulation* 100(4)**,** 393-399. doi: doi:10.1161/01.CIR.100.4.393.

Pincus, S.M., Gladstone, I.M., and Ehrenkranz, R.A. (1991). A regularity statistic for medical data analysis. *Journal of Clinical Monitoring* 7(4)**,** 335-345. doi: 10.1007/BF01619355.

Sassi, R., Cerutti, S., Lombardi, F., Malik, M., Huikuri, H.V., Peng, C.-K., et al. (2015). Advances in heart rate variability signal analysis: joint position statement by the e-Cardiology ESC Working Group and the European Heart Rhythm Association co-endorsed by the Asia Pacific Heart Rhythm Society. *EP Europace* 17(9)**,** 1341-1353. doi: 10.1093/europace/euv015.

Xiao, M., Yan, H., Yang, X., Li, Y., and Zhu, R. (Year). "Multiscale entropy based analysis of HRV during sleep", in: *2012 5th International Conference on BioMedical Engineering and Informatics*), 558-562.
